# An integrated framework for identifying clinical-laboratory indicators for novel pandemics: COVID-19 and MIS-C

**DOI:** 10.1038/s41746-021-00547-9

**Published:** 2022-01-20

**Authors:** Adam D. Nahari, Mary Beth F. Son, Jane W. Newburger, Ben Y. Reis

**Affiliations:** 1 Predictive Medicine Group, Computational Health Informatics Program, Boston Children’s Hospital, Boston, MA USA; 2 Division of Immunology, Boston Children’s Hospital, Boston, MA USA; 3 Harvard Medical School, Boston, MA USA; 4 Department of Cardiology, Boston Children’s Hospital, Boston, MA USA

**Keywords:** Epidemiology, Viral infection, Translational research

## Abstract

During the critical early stages of an emerging pandemic, limited availability of pathogen-specific testing can severely inhibit individualized risk screening and pandemic tracking. Standard clinical laboratory tests offer a widely available complementary data source for first-line risk screening and pandemic surveillance. Here, we propose an integrated framework for developing clinical-laboratory indicators for novel pandemics that combines population-level and individual-level analyses. We apply this framework to 7,520,834 clinical laboratory tests recorded over five years and find clinical-lab-test combinations that are strongly associated with SARS-CoV-2 PCR test results and Multisystem Inflammatory Syndrome in Children (MIS-C) diagnoses: Interleukin-related tests (e.g. IL4, IL10) were most strongly associated with SARS-CoV-2 infection and MIS-C, while other more widely available tests (ferritin, D-dimer, fibrinogen, alanine transaminase, and C-reactive protein) also had strong associations. When novel pandemics emerge, this framework can be used to identify specific combinations of clinical laboratory tests for public health tracking and first-line individualized risk screening.

## Introduction

During the critical early stages of an emerging pandemic, the availability of pathogen-specific testing is often severely limited due to the considerable time necessary for developing, refining, manufacturing, scaling, and distributing novel pathogen-specific tests^1^. This scarcity can significantly inhibit pandemic tracking and individual risk screening, both critical public health measures during the early stages of a pandemic^2–4^.

While PCR testing has emerged as the current leading approach for SARS-CoV-2 testing^2,3,5,6^, there were widespread shortages and delays in its availability during the first few months of the COVID-19 pandemic. Testing for children was especially limited and difficult to procure, as the few available tests were prioritized for adult patients. Many testing sites in the United States, including those run by cities and states, did not test any children or set age minimums that excluded young children^7^. Although children faced a lower risk of serious illness and death from SARS-CoV-2 infection than adults, testing of children was important as a new, severe illness afflicting children emerged in the Spring of 2020 which became known as Multisystem Inflammatory Syndrome in Children (MIS-C)^8–11^. Furthermore, multiple studies indicated that children played an important role in spreading the SARS-CoV-2 infection^12–15^, including through schools^16^.

With pathogen-specific testing in short supply, standard clinical laboratory tests offer a potential complementary source of information during the early stages of a novel pandemic. These tests are both widely available and easily scalable to meet demand, and generally provide faster results than novel pathogen-specific tests such as those based on PCR. Routine clinical laboratory tests could potentially be used both as statistical indicators of population-level spread, as well as first-line screening tools to most efficiently allocate the limited supply of pathogen-specific tests and identify individuals at risk for severe illness.

Laboratory tests can also help in the later stages of a pandemic. Even now, over one year into the COVID-19 pandemic, diagnosing MIS-C is difficult, as it is defined by broad clinical criteria^17^, including features that overlap with other conditions such as septic shock. Laboratory tests, and specific combinations of laboratory tests, have the potential to be useful in tracking and assessing individual risk of MIS-C.

In this study, we propose an integrated framework for developing clinical laboratory-based indicators for novel pandemics and apply it to SARS-CoV-2 infection and MIS-C in a pediatric setting. The proposed integrated framework combines both population-level and individual-level approaches in order to identify the most useful laboratory test combinations for pandemic tracking and risk-screening.

## Results

Supplementary Figure 1 presents a data flow chart describing the populations used for the analyses. Table 1 shows the number of confirmed SARS-CoV-2 infections and the number of confirmed MIS-C cases per month at BCH from January 2020 through October 2020. According to these data, the first wave of SARS-CoV-2 infections peaked in April 2020, and the first wave of MIS-C diagnoses peaked in May 2020.

**Table 1:** Confirmed SARS-CoV-2 infections and MIS-C diagnoses.

Month	SARS-CoV-2 PCR unique patients tested	SARS-CoV-2 PCR unique patients first positive result	SARS-CoV-2 PCR positivity rate (%)	MIS-C unique patients diagnosed
2020-01	0	0	–	0
2020–02	0	0	–	0
2020–03	143	6	4.2	2
2020–04	1106	70	6.3	5
2020–05	2446	58	2.3	22
2020–06	3371	17	0.5	9
2020–07	3869	40	1	9
2020–08	4223	51	1.2	3
2020–09	4076	62	1.5	1
2020–10	5350	148	2.8	1

Unique confirmed SARS-CoV-2 infections and confirmed MIS-C diagnoses at Boston Children’s Hospital, by month, January 2020 through October 2020.

**Fig. 1:**
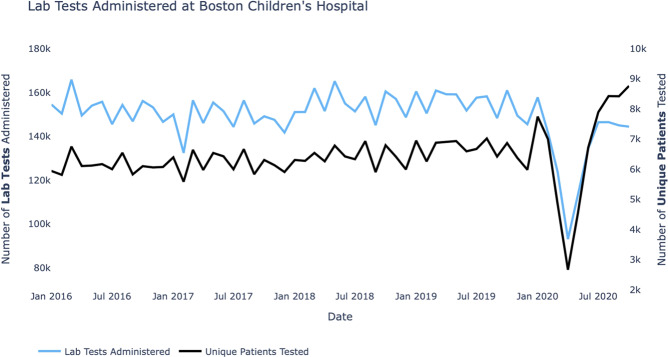
Healthcare utilization during the COVID-19 pandemic. Monthly laboratory tests administered and unique patients receiving at least one test at Boston Children’s Hospital from January 2016 through October 2020.

From January 2016 through October 2020, a total of 7,520,834 test results belonging to 38 clinical laboratory test types were recorded at BCH for all patients under 19 years old. These laboratory tests were administered to 143,569 individuals and grouped into 806,294 episodes of care. Figure 1 shows the number of clinical laboratory tests administered and the number of unique patients who received at least one clinical laboratory test at BCH each month. A clear decline in both of these monthly rates is visible around March 2020, consistent with the drop in hospital utilization due to the initial lockdown measures and elevated public caution during the early stages of the COVID-19 pandemic.

### Population approach

**Fig. 2:**
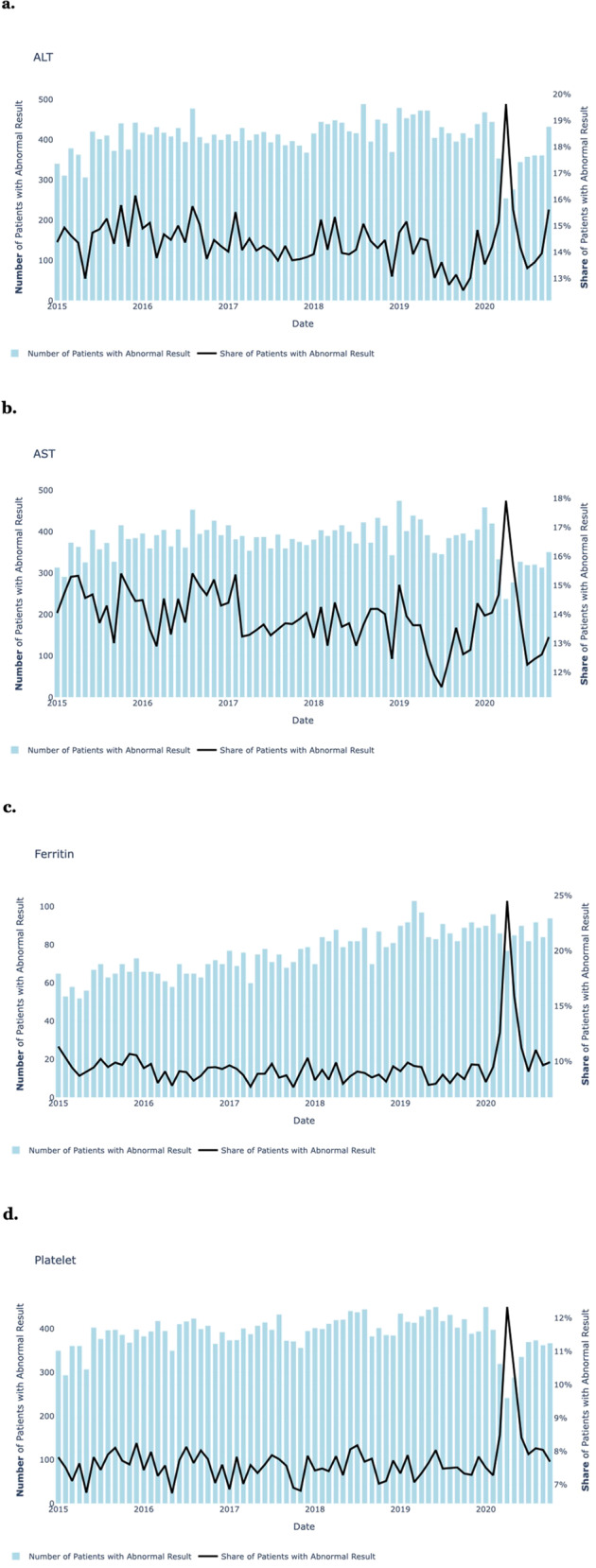
Population approach, single laboratory tests. The total number of monthly abnormal test results (blue bars) and test abnormality rates (black line) recorded from January 2016 through August 2020, for selected single clinical laboratory tests: **a** ALT (alanine aminotransferase), **b** AST (aspartate aminotransferase), **c** ferritin, and **d** platelets.

We began by examining the results of single clinical laboratory tests over time. Figure 2 shows monthly abnormal counts and abnormal rates for selected clinical laboratory tests from January 2016 through October 2020. For almost all tests (neutrophils, lymphocytes, eosinophils, basophils, hematocrit, hemoglobin, red blood count, platelets, international normalized ratio, fibrinogen, C-reactive protein, erythrocyte sedimentation rate, alanine transaminase, aspartate transaminase, albumin, gamma-glutamyltransferase, bilirubin, and blood urea nitrogen), the absolute number of abnormal tests declined during the initial peak of the pandemic in April and May of 2020, while the abnormality rate went up (e.g. for Ferritin, the abnormality rate increased from below 10% to almost 25%). This is consistent with lower overall hospital utilization combined with a higher severity patient case-mix, as patients with less severe conditions were more inclined to stay home during the early stages of the pandemic or seek care through other channels, while more severely ill patients continued to seek care at BCH. While the sharp spikes in abnormal rates are noteworthy, given the large underlying shifts in case-mix it can be difficult to deduce any conclusions based solely on a historical analysis of these single tests.

**Fig. 3:**
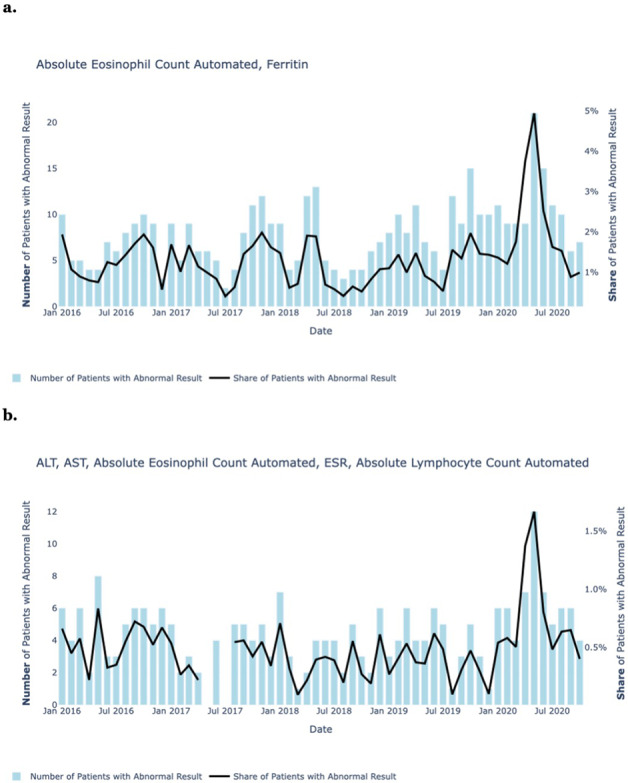
Population approach, laboratory test combinations. The total number of monthly abnormal test results (blue bars) and test abnormality rates (black line) recorded from January 2016 through August 2020, for selected combinations of clinical laboratory tests: **a** eosinophil and ferritin; **b** ALT, AST, eosinophil, ESR, and lymphocyte.

Next, we examined combinations of clinical laboratory tests over time. Figure 3 shows monthly abnormal counts and abnormal rates for selected combinations of clinical laboratory tests from January 2016 through August 2020. Unlike with single tests, for certain combinations of tests (e.g. eosinophils and ferritin), both the share of patients with abnormal results and the absolute number of patients with abnormal results went up during the peak of the pandemic: certain combinations of clinical laboratory tests not only exhibit abnormality rates that are highly correlated with COVID-19 rates over time but also increase in absolute terms along with COVID-19 rates, despite drops in healthcare utilization.

**Fig. 4:**
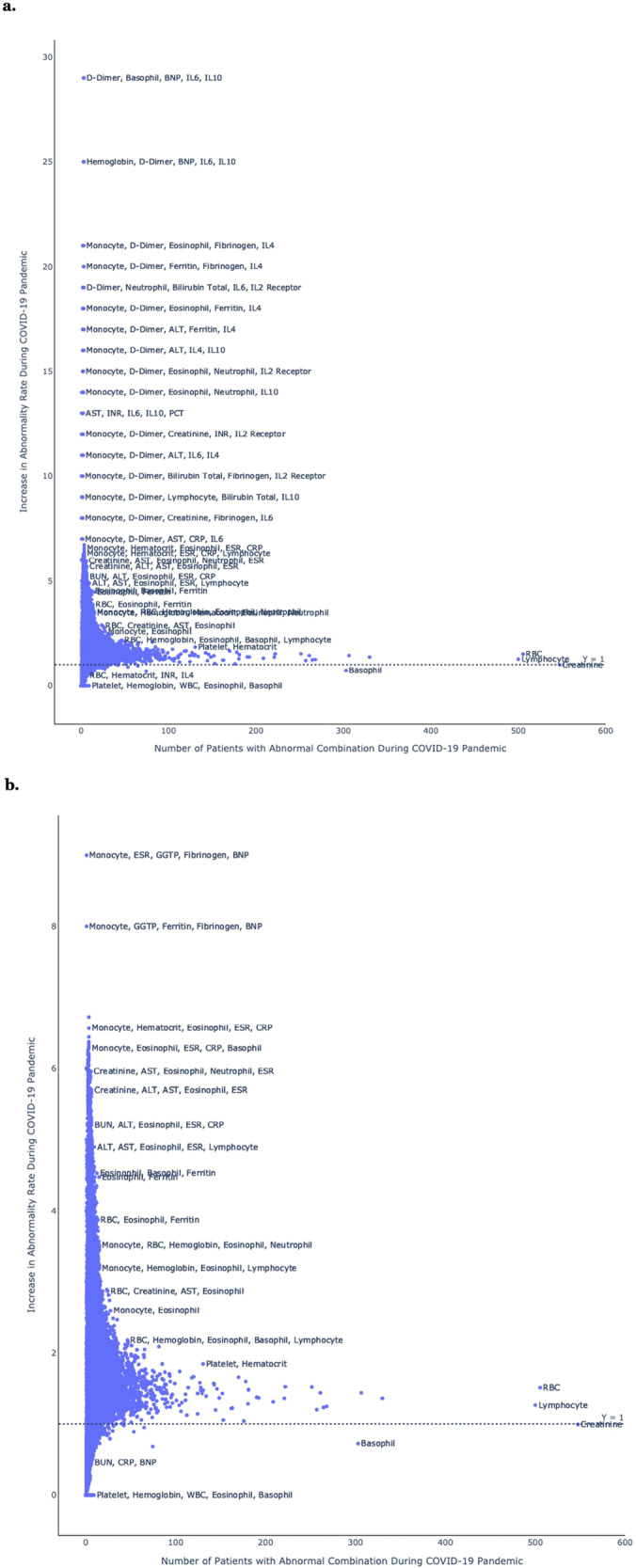
Population approach, overview. Systematic comparison of all laboratory test combinations observed during the COVID pandemic, according to the number of abnormal test results (X-axis), and the percentage increase in test abnormality rates relative to historical baseline (Y-axis). Results are shown **a** with Interleukin (IL) tests and **b** without Interleukin (IL) tests.

Systematically comparing all observed tests and test combinations, Fig. 4 reveals certain high-volume single laboratory tests (e.g. creatinine, lymphocyte, RBC) that had a high number of abnormal test results observed during the COVID-19 pandemic but little change in test abnormality rates, and certain less common combinations of tests (e.g. those including monocyte, D-dimer, fibrinogen, ESRR, BNP, and IL2/IL4/IL6) that had a low number of abnormal test results observed during the COVID-19 pandemic but a high percentage increase in test abnormality rates.

While the results from the population approach suggest that there is an association between certain clinical laboratory test combinations and SARS-CoV-2 infection, these are population-level findings, and thus subject to the inherent limitations discussed below.

### Individual approach

Next, we examined individual-level associations between clinical laboratory test combinations and SARS-CoV-2 and MIS-C status. During the study period, 24,587 individuals at BCH were tested for SARS-CoV-2, of whom 452 (1.84%) were found to be PCR-positive. Among the individuals tested for SARS-CoV-2, 9,076 also had at least one clinical laboratory test recorded, and 188 (2.07%) of those were PCR-positive. Having at least one clinical laboratory test recorded was associated with a slightly elevated PCR-positivity rate. During the study period, 52 individuals at BCH were diagnosed with MIS-C.

**Fig. 5:**
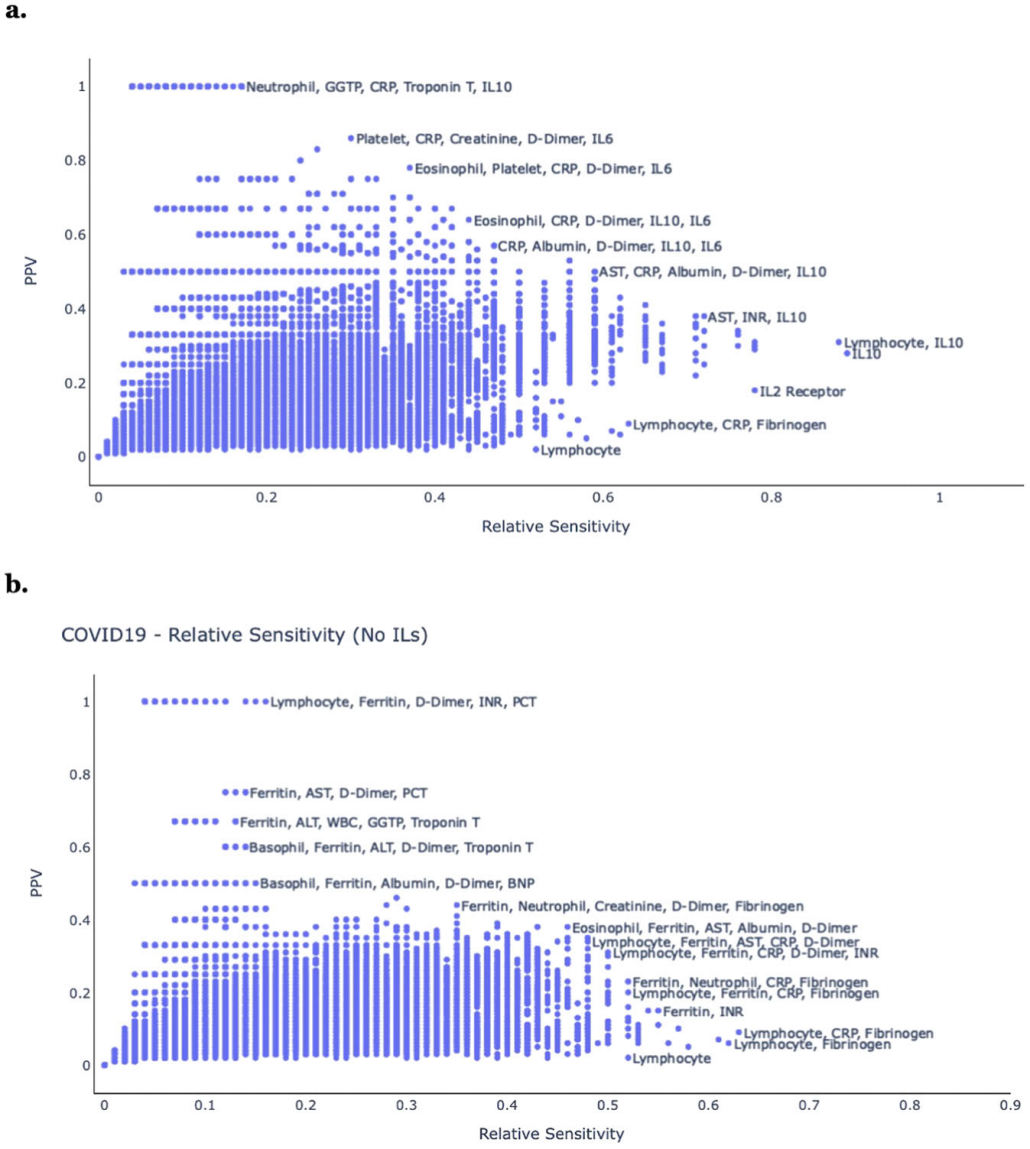
Individual approach, SARS-CoV-2. Comparison of different laboratory test combinations, according to the relative sensitivity (X-axis) and the PPV (Y-axis) for association with SARS-Cov-2 infection status. Results are shown **a** with IL tests and **b** without IL tests.

We systematically compared the results of each test combination to PCR-confirmed SARS-CoV-2 infection status and MIS-C diagnosis for each individual. For each test combination, we calculated the PPV, relative sensitivity, and absolute sensitivity for SARS-CoV-2 infection. Figure 5 shows a general tradeoff between PPV and relative sensitivity for different test combinations relating to COVID-19. The IL-related tests had the strongest associations with SARS-CoV-2 infection. Combinations with the highest PPV included {neutrophil, GGTP, C-reactive protein, troponin T, IL10} and {platelets, C-reactive protein, creatinine, D-dimer, IL6}. Excluding IL-related tests, which are not readily available at all medical centers, combinations with the highest PPV included {lymphocyte, ferritin, D-dimer, INR, PCT} and {ferritin, AST, D-dimer, PCT}. Combinations with the highest relative sensitivity included {lymphocyte, IL10} and {AST, INR, IL10}. Excluding IL-related tests, combinations with the highest relative sensitivity included {lymphocyte, C-reactive protein, fibrinogen} and {ferritin, INR}.

The combinations of {AST, C-reactive protein, albumin, D-dimer, IL10} and {C-reactive protein, albumin, D-dimer, IL10, IL6} provided a balance between PPV and relative sensitivity. Excluding IL-related tests, the combinations of {eosinophil, ferritin, AST, albumin, D-dimer} and {lymphocyte, ferritin, AST, C-reactive protein, D-dimer} provided a balance between PPV and relative sensitivity. Certain clinical laboratory tests, including C-reactive protein, ferritin, D-dimer, PCT, IL10, lymphocyte, AST, and albumin appear in many of these combinations, consistent with their singleton results, indicating that they are good candidates for clinical screening use - depending on the specific needs and test-availabilities of the particular clinical setting.

Supplementary Figure 2 is similar to Fig. 5, but shows absolute sensitivity instead of relative sensitivity. Supplementary Figure 3 shows the effects of increasing test combination size – from single tests to five-test combinations: the greater number of labs in a combination, the greater the PPV and relative sensitivity.

**Fig. 6:**
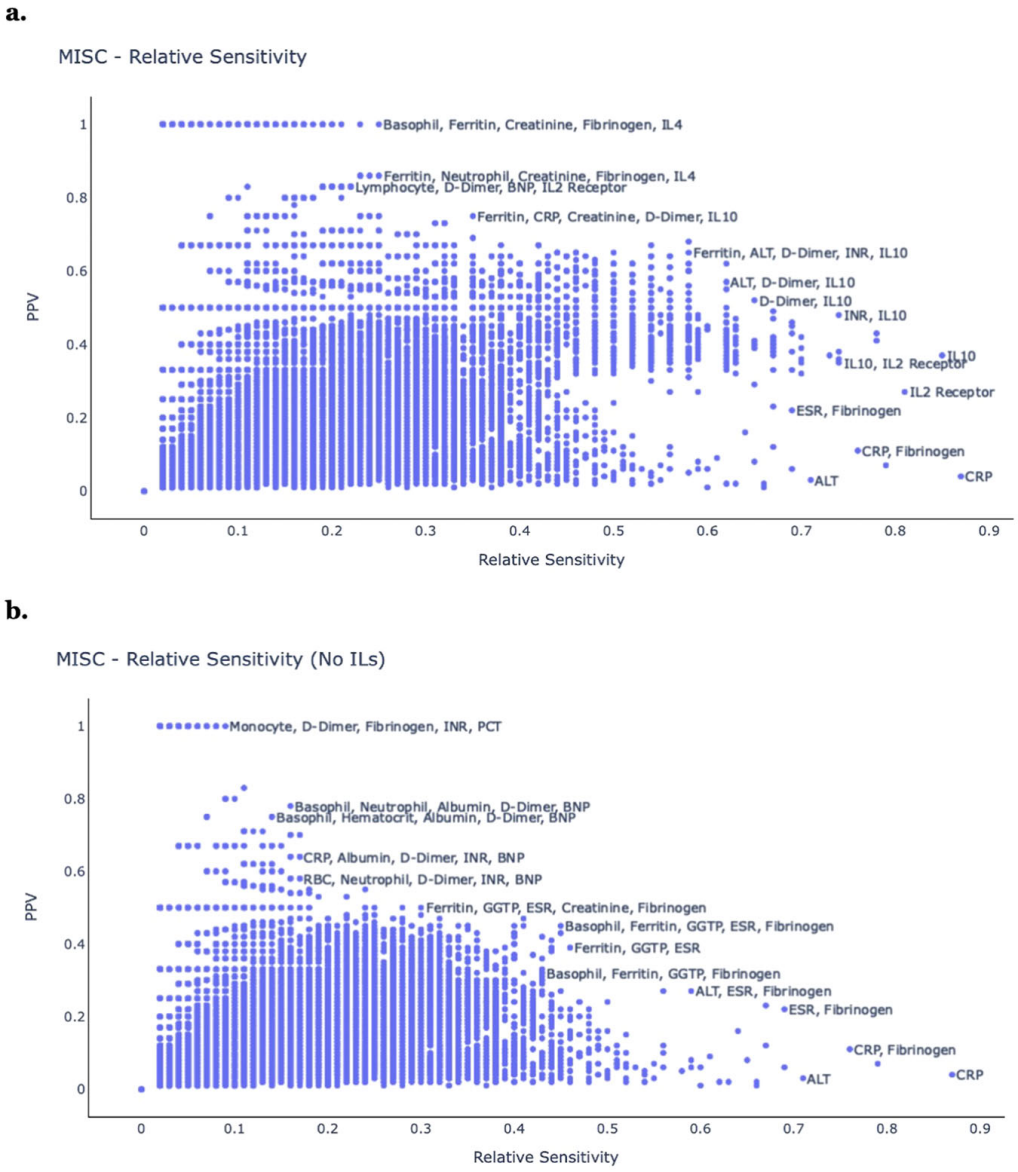
Individual approach, MIS-C. Comparison of different laboratory test combinations, according to the relative sensitivity for MIS-C (X-axis), and the PPV (Y-axis). Results are shown **a** with IL tests and **b** without IL tests.

Next, we examined MIS-C. For each test combination, we calculated the PPV, relative sensitivity, and absolute sensitivity for MIS-C. Figure 6 shows a general tradeoff between PPV and relative sensitivity for different test combinations relating to MIS-C. Combinations with the highest PPV included {basophil, ferritin, creatinine, fibrinogen, IL4} and {ferritin, neutrophil, creatinine, fibrinogen, IL4}. Excluding IL-related tests, combinations with the highest PPV included {monocyte, D-dimer, fibrinogen, INR, PCT} and {basophil, neutrophil, albumin, D-dimer, BNP}. Combinations with the highest relative sensitivity included {C-reactive protein, fibrinogen} and {IL10, IL2 receptor}. Excluding IL-related tests, the combinations with the highest relative sensitivity included {C-reactive protein, fibrinogen} and {ALT, ESR, fibrinogen}.

The combinations of {ferritin, ALT, D-dimer, INR, IL10} and {ALT, D-dimer, IL10} balanced PPV and relative sensitivity. Excluding IL-related tests, the combinations of {ferritin, GGTP, ESR, creatinine, fibrinogen} and {basophil, ferritin, GGTP, ESR, fibrinogen} balanced PPV and relative sensitivity. Certain clinical laboratory tests, including ferritin, fibrinogen, basophil, creatinine, and D-dimer, appear in many of these combinations, consistent with their singleton results, indicating that they are good candidates for clinical screening use - depending on the specific needs and test-availabilities of the particular clinical setting. Supplementary Figure 4 is similar to Fig. 6, but shows absolute sensitivity instead of relative sensitivity.

### Comparison of population and individual approaches

We compared the top results of both population and individual approaches for SARS-CoV-2 and MIS-C. Certain laboratory tests appeared in many of the top-performing combinations in both approaches, including IL10, IL2 receptor, hemoglobin, monocytes, eosinophils, basophils, ferritin, C-reactive protein, ESR, D-dimer, troponin, AST, and ALT. Others appeared in many of the top-performing combinations in only one approach, such as IL6, IL4, fibrinogen, neutrophils, and B type-natriuretic protein (BNP) for the population approach, and lymphocytes, INR, creatinine, and albumin for the individual approach.

Supplementary Figure 5 is similar to Fig. 5 but is filtered to show only those laboratory test combinations that peaked in April or May, thus presenting the clinical laboratory test combinations that were identified by both the population-based and individual-based approaches. Supplementary Figure 6 is similar to Fig. 6 but filtered to show only those laboratory test combinations that peaked in April or May, thus presenting the clinical laboratory test combinations that were identified by both the population-based and individual-based approaches. Supplementary Figures 7–9 are similar to Figs. 4–6, but with different thresholds for minimal sample size.

## Discussion

We propose an integrated framework for developing clinical-laboratory-based indicators of novel pandemics. Applying this framework to a large healthcare setting, we found specific clinical laboratory test combinations that are associated with SARS-CoV-2 infection and MIS-C diagnosis. Combinations of clinical laboratory tests were more closely associated with the outcomes than individual clinical laboratory tests, and the specificity of the association increased with the number of tests in the combination. There were similarities in the laboratory test combinations identified by both the population and individual-based approaches. For example, test combinations containing IL-10, ferritin or D-dimer showed strong associations in both approaches.

The association with laboratory test combinations was stronger with MIS-C than with SARS-CoV-2 infection. While SARS-CoV-2 tests are today available in many parts of the world, identifying MIS-C patients is still difficult as it is defined by broad clinical criteria^17^, including features that overlap with other conditions such as septic shock. Our results indicate that specific clinical laboratory test combinations could be used as indicators of MIS-C in a pediatric patient presenting with severe illness, especially in a setting that is currently experiencing elevated SARS-CoV-2 prevalence.

This integrated framework, combining both population and individual level approaches, can be used as a complementary method for characterizing novel pandemics, especially during their critical early phases when pathogen-specific tests are not yet widely available. Clinical laboratory test combinations could potentially be used for two purposes: (1) Tracking population-level trends in the prevalence and spread of the pandemic across the population; (2) First-line individual screening to prioritize the allocation of limited pathogen-specific tests, and to identify high-risk individuals for the purposes of infection control, PPE requirements, patient isolation, and treatment.

Our analysis revealed a tradeoff between sensitivity and PPV for different test combinations. There was also a tradeoff between the number of patients tested for a given combination and PPV. In light of these tradeoffs, the choice of optimal test combinations depends on the intended purpose. For individual risk screening, there would likely be a preference for maximizing sensitivity in order to avoid missed cases. In this study, the highest sensitivity combinations included IL4 and IL10. Although these tests are less widely available and may involve longer wait times for test processing, their use could potentially be scaled up to screen additional patients of interest. On the other hand, for the purposes of population surveillance, there would likely be a preference for maximizing specificity and PPV, since the goal is not to catch every case, but rather to develop a reliable indicator of cases rising or falling. As such, commonly available laboratory tests may be preferable.

A few studies have examined the use of routine clinical laboratory tests for the diagnosis of SARS-CoV-2 infection, primarily focused on the adult population. Yang et al.^18^ used machine learning along with 27 types of routine clinical laboratory tests to predict an individual’s SARS-CoV-2 infection status up to two days before they received a positive pathogen-specific PCR test. The same authors also found that patients with higher viral loads had a stronger association between the clinical test profile and a positive SARS-CoV-2 PCR result^19^.

Santotoribio et al.^20^ found that lymphocytes, eosinophils, D-dimer, LDH, high sensitivity C-reactive protein, and ferritin can be used to differentiate adult patients with and without COVID-19. Another study developed a risk score that included routine laboratory parameters (C-reactive protein, lactate dehydrogenase, ferritin, absolute neutrophil, and lymphocyte counts), along with demographic data and chest X-ray/CT results to assess SARS-CoV-2 infection in an Emergency Department^21^. Stegeman et al.^22^ found abnormal results from IL-6, C-reactive protein, and lymphocyte lab tests to be sensitive in predicting COVID-19 infection. Other studies focused on predicting positive SARS-CoV-2 infection using clinical features, simple blood test data, and baseline health such as age, past medical history, and vital sign abnormalities^23–26^. Studies have also been performed to analyze the relationships between tests and severe outcomes^27–36^ or death^37^ for patients already diagnosed with SARS-CoV-2 infection or COVID-19. Some studies looked specifically at pediatric patients^38^ and at MIS-C^8,9,39–42^ and found specific sets of findings associated with SARS-CoV-2 infection and MIS-C.

To the best of the authors’ knowledge, this is the first study examining both population-level and individual-level approaches to determining the optimal combinations of tests, and to provide a systematic understanding of the tradeoffs of different test combinations. It is also the first to focus on identifying laboratory test combinations for assessing SARS-CoV-2 infection and MIS-C in a pediatric population. As mentioned above, this is important since the limited number of SARS-CoV-2 tests that were available at the start of the pandemic were often reserved for adults, despite children also being able to spread the pandemic. This study also provides a general integrated framework for identifying appropriate laboratory test combinations for novel pandemics, including during the early stages of the pandemic when pathogen-specific tests are scarce or unavailable.

This study has a number of limitations. First, it was conducted at a single major tertiary healthcare center, and while we would expect the framework to be generalizable to other sites, future work includes applying this framework to other sites, demographic groups, and pandemics. Additional limitations for this study revolve around the SARS-CoV-2 PCR test: False positives and negatives may occur that could impact the analysis through introducing noise to the measured signals. Additionally, PCR tests were only performed on patients suspected of having SARS-CoV-2 infection, which may create bias. The lab tests used in this study – aside from limited IL tests – are widely performed each year at BCH and other academic medical centers. In order for the proposed framework to be implemented elsewhere, a site would only need access to the relevant clinical data.

Each of the two approaches used in this study has strengths and weaknesses: the population approach has inherent limitations, as it does not measure a direct connection between test combinations and SARS-CoV-2 and MIS-C status in individual people, but rather relies on population-level associations over time. It may, therefore, be subject to potential sources of bias and confounding. For example, hospital case-mix may change during a pandemic, which could affect the number of laboratory tests of each type that are administered, as well as their rates of abnormal results. Changes in case-mix may be due to shifts in health-seeking behaviors or psychological or physiological responses to the pandemic or to pandemic mitigation measures such as closures and lockdowns. Additionally, the change in health-seeking behavior, the lower levels of RSV and other common viruses circulating in the population, and the frequent and varied use of adjunctive treatment with biologics, second doses of IVIG or glucocorticoids also added potential sources of noise to the data. While other types of conditions such as septic shock or sepsis could have also impacted the laboratory test data recorded during the pandemic, the population-approach proposed here compares the data during the pandemic to baseline data recorded during the five years prior to the pandemic, which should have captured the baseline levels of these other conditions. As seen in Fig. 2, in some cases (e.g. ferritin) the number of patients with abnormal results increased over time – this could be due to increases in the patient population over time. The population-approach examines the abnormality percentage over time, so should be robust to these long-term trends.

While the individual approach assesses the association between test results and SARS-CoV-2 and MIS-C status more directly, it relies on cross-referencing with pathogen-specific tests, so its feasibility may be limited when pathogen-specific tests are not widely available. The population approach may thus be the only method available in the early stages of an emerging pandemic. In this study, the population approach provided results that were very similar to the individual-level approach, indicating that it provides substantial value despite its limitations.

In summary, our findings demonstrate that laboratory test combinations can serve as useful complementary data sources for tracking and risk screening for SARS-CoV-2 infection and MIS-C in a pediatric setting. The proposed general framework can also be used by clinicians and scientists worldwide to respond to future emerging pandemics, revealing additional properties of pathogens and their disease course.

## Methods

### Data retrieval

We examined data on all patients younger than 19 years old seen at Boston Children’s Hospital (BCH) between 1 January 2016 and 31 October 2020 (Table 2). We included data on 38 types of clinical laboratory tests (Table 3), SARS-CoV-2 PCR test results, and physician-confirmed MIS-C diagnoses according to the criteria defined by the Centers for Disease Control (CDC)^43^.

**Table 2:** Characteristics of the patient population.

Total number of patients	143,569
Median age (IQR) — yr	9 (3–14)
Age group — no. (%)	
0–5 yr	52,059 (36%)
6–10 yr	27,204 (19%)
11–15 yr	33,059 (23%)
16–19 yr	31,247 (22%)
Sex — no. (%)	
Female	73,117 (51%)
Male	70,452 (49%)

**Table 3:** Clinical laboratory tests with normal ranges.

Clinical laboratory test	Abnormality threshold	Max/min
Absolute eosinophil count	<0.02 × 10^9^ cells/L	Min
Absolute lymphocyte count	<1.5 × 10^9^ cells/L in patients 8 months of age or older and of <4.5 × 10^9^ cells/L in patients younger than 8 months of age	Min
Albumin	≤3 g/dl	Min
ALT	≥40 U/L	Max
AST	>50 U/L	Max
Basophil automated	<0.021 × 10^9^ cells/L	Min
Bilirubin, direct	>0.4 mg/dL	Max
Bilirubin, total	>1 mg/dL	Max
BNP	>400 pg/mL	Max
BUN	>18 mg/dL	Max
C-Reactive Protein	≥3 mg/L	Max
Creatinine	>0.59 mg/dL	Max
D-Dimer	>300 ng/mL	Max
ESR	≥40 mm/h	Max
Ferritin	>500 mg/L	Max
Fibrinogen	>400 mg/dL	Max
GGTP	>51 U/L	Max
Hematocrit	<30 percent	Min
Hemoglobin	<9 g/dL	Min
IL10	>9.2 pg/mL	Max
IL2	>9 pg/mL	Max
IL2 Receptor	>858.2 pg/mL	Max
IL4	>3 pg/mL	Max
IL6	>9 pg/mL	Max
IL8	>116 pg/mL	Max
INR	>1.1	Max
LDH	>500 U/L	Max
Monocyte automated	<0.2 × 10^9^ cells/L	Min
Neutrophil automated	>7.7 × 10^9^ cells/L	Max
PCT	>50 ng/mL	Max
Platelet	<150,000 mm^3^	Min
RBC	<3.8 × 10^12^ cells/L	Min
TNF	>22 pg/mL	Max
Troponin I	>0.04 ng/mL	Max
Troponin T	>0.1 ng/mL	Max
WBC	>20 × 10^9^ cells/L	Max

Ranges for 38 clinical laboratory tests examined in the study, including blood counts: WBC, hemoglobin, hematocrit, RBC, platelets, neutrophil, ALC, monocyte, basophil, AEC; inflammatory markers: ESR, C-reactive protein, procalcitonin, ferritin; coagulation markers: fibrinogen, D dimer, INR; liver function tests: AST, ALT, albumin, bilirubin, GGTP, LDH; cardiac injury markers: troponin, BNP; chemistries: BUN, creatinine; cytokine panel/interleukins: IL’s and TNF. Each test is shown with the threshold used to determine abnormality. (Min: low values are of interest; max: high values are of interest).

For each patient, we grouped lab tests administered within 7 days of one another into the same “episode of care.” (Some episodes of care extended for longer than 7 days since they were “chained” together by intermediate labs. For example, if a patient received a lab test on 1 January 2016, a second lab test on 5 January 2016, and a third lab test on 10 January 2016, those three lab tests were grouped into the same episode of care that began on 1 January 2016 and ended on 10 January 2016). Each test result was categorized as either normal or abnormal based on the criteria listed in Table 3. For each patient, episode of care, and lab test type, we examined either the maximum or minimum result – depending on which test direction indicated an abnormality of concern, as indicated in Table 3. Supplementary Table 1 shows the number of tests performed in 2020, by test type and age group.

### Data analysis: population approach

We examined historical trends for the 38 clinical laboratory test types included in this study, as well as all possible combinations of between two and five of these laboratory tests (e.g. {D-dimer, Ferritin} is one possible combination consisting of two tests). For each test combination in each month from January 2016 to October 2020, we calculated the number of patients with abnormal lab test results, as well as the share of patients with abnormal lab test results among all the patients tested for the particular combination. Each combination was assigned to a month based on the date of the first lab test recorded as part of the combination.

In order to assess the increase in abnormality rate during the pandemic, we calculated the ratio of the abnormal rate during the COVID-19 pandemic to the abnormal rate during the historical baseline. We identified combinations of tests for which abnormal rates changed the most during the COVID-19 pandemic relative to historical baselines.

### Data analysis: individual approach

We analyzed the individual-level associations between the results of clinical laboratory test combinations and SARS-CoV-2 PCR test results, as well as with physician-confirmed diagnoses of MIS-C. For each episode of care, we determined whether a positive SARS-CoV-2 PCR test was recorded between 7 days before and 7 days after the episode of care. We also determined whether an MIS-C diagnosis was recorded between seven days before and seven days after the episode of care. For each test combination, we calculated the following metrics to systematically identify optimal combinations of laboratory testing:

PPV - among individuals who were tested both for SARS-CoV-2 (PCR) and for the clinical laboratory test combination, of those who received an abnormal result for all the tests in the lab test combination, what percentage were also positive for SARS-CoV-2 infection?

Relative sensitivity - among individuals tested both for SARS-CoV-2 (PCR) and for the clinical laboratory test combination, of those who tested positive for SARS-CoV-2 infection, what percentage also had abnormal results for all the tests in the lab test combination?

Absolute sensitivity - among all individuals who tested positive for the SARS-CoV-2 infection (regardless of whether or not they were tested for the test combination), what percentage also had abnormal results for the lab test combination?

We also calculated each of the above three metrics for MIS-C diagnosis.

### Ethics

This study was approved by the Boston Children’s Hospital Institutional Review Board. Informed consent was not required as the research involved analysis of aggregated de-identified data.

### Reporting summary

Further information on research design is available in the Nature Research Reporting Summary linked to this article.

## Supplementary information

Supplementary Materials

Reporting Summary

## Data Availability

Other than the aggregate statistics reported in the manuscript, which are available from the author upon request, the data analyzed for this study is restricted due to medical privacy regulations.

## Code availability

The Code used for the analysis is available at 10.5281/zenodo.5668279.
