# Supplementary material for: An integrated framework for identifying clinical-laboratory indicators for novel pandemics: COVID-19 and MIS-C
Source: NPJ Digit Med. 2022 Jan 20;5:9. doi: 10.1038/s41746-021-00547-9 (PMC8776774; doi:10.1038/s41746-021-00547-9)

Supplementary Materials

An Integrated Framework for Identifying Clinical-Laboratory Indicators for Novel Pandemics: COVID-19 and MIS-C

Adam D. Nahari[1], Mary Beth F. Son, MD[2,4], Jane W. Newburger, MD MPH[3,4], Ben Y. Reis, PHD[1,4]

[1] *Predictive Medicine Group, Computational Health Informatics Program, Boston Children's Hospital, Boston, MA*

[2] *Division of Immunology, Boston Children's Hospital, Boston, MA*

[3] *Department of Cardiology, Boston Children's Hospital, Boston, MA*

[4] *Harvard Medical School, Boston, MA*

Supplementary Table 1. Number of lab tests performed in 2020, by test type and age group.

Lab Test	Age group 0 - 5 yr	Age group 6 - 10 yr	Age group 11 - 15 yr	Age group 16 - 19 yr	Total
ALT	12,645	8,293	10,260	10,540	41,738
AST	12,038	8,209	9,986	10,186	40,419
Absolute Eosinophil Count	12,595	9,666	12,121	12,238	46,620
Absolute Lymphocyte Count	12,595	9,666	12,121	12,238	46,620
Albumin	12,878	7,621	9,053	9,555	39,107
BNP	775	256	276	303	1,610
BUN	30,812	13,942	16,784	16,132	77,670
Basophil Automated	12,595	9,666	12,121	12,238	46,620
Bilirubin, Direct	12,930	7,513	8,659	8,404	37,506
Bilirubin, Total	13,178	7,575	8,730	8,519	38,002
C-Reactive Protein	5,798	3,775	5,176	6,384	21,133
Creatinine	30,968	14,045	16,887	16,086	77,986
D-Dimer	940	430	332	620	2,322
ESR	2,613	2,711	4,269	5,533	15,126
Ferritin	2,846	1,800	2,221	2,611	9,478
Fibrinogen	3,834	895	824	913	6,466
GGTP	2,165	1,094	1,541	1,900	6,700
Hematocrit	31,255	15,647	17,438	17,581	81,921
Hemoglobin	31,339	15,496	17,210	17,356	81,401
IL10	86	42	43	89	260
IL2	85	42	43	88	258
IL2 Receptor	84	42	43	91	260
IL4	85	42	43	89	259
IL6	92	44	44	92	272
IL8	85	42	44	88	259
INR	6,736	2,120	2,316	2,515	13,687
LDH	2,801	1,995	2,123	1,831	8,750
Monocyte Automated	12,595	9,666	12,121	12,238	46,620
Neutrophil Automated	12,594	9,666	12,121	12,238	46,619
PCT	2,257	569	559	582	3,967
Platelet	30,484	15,504	17,158	17,168	80,314
RBC	30,513	15,439	17,157	17,181	80,290
TNF	85	43	43	89	260

Troponin I	523	172	224	342	1,261
Troponin T	32	2	1	2	37
WBC	30,498	15,428	17,139	17,150	80,215
**Total**	**374,434**	**209,158**	**247,231**	**251,210**	**1,082,033**

Supplementary Figure 1. Flow Chart of population used for analysis.

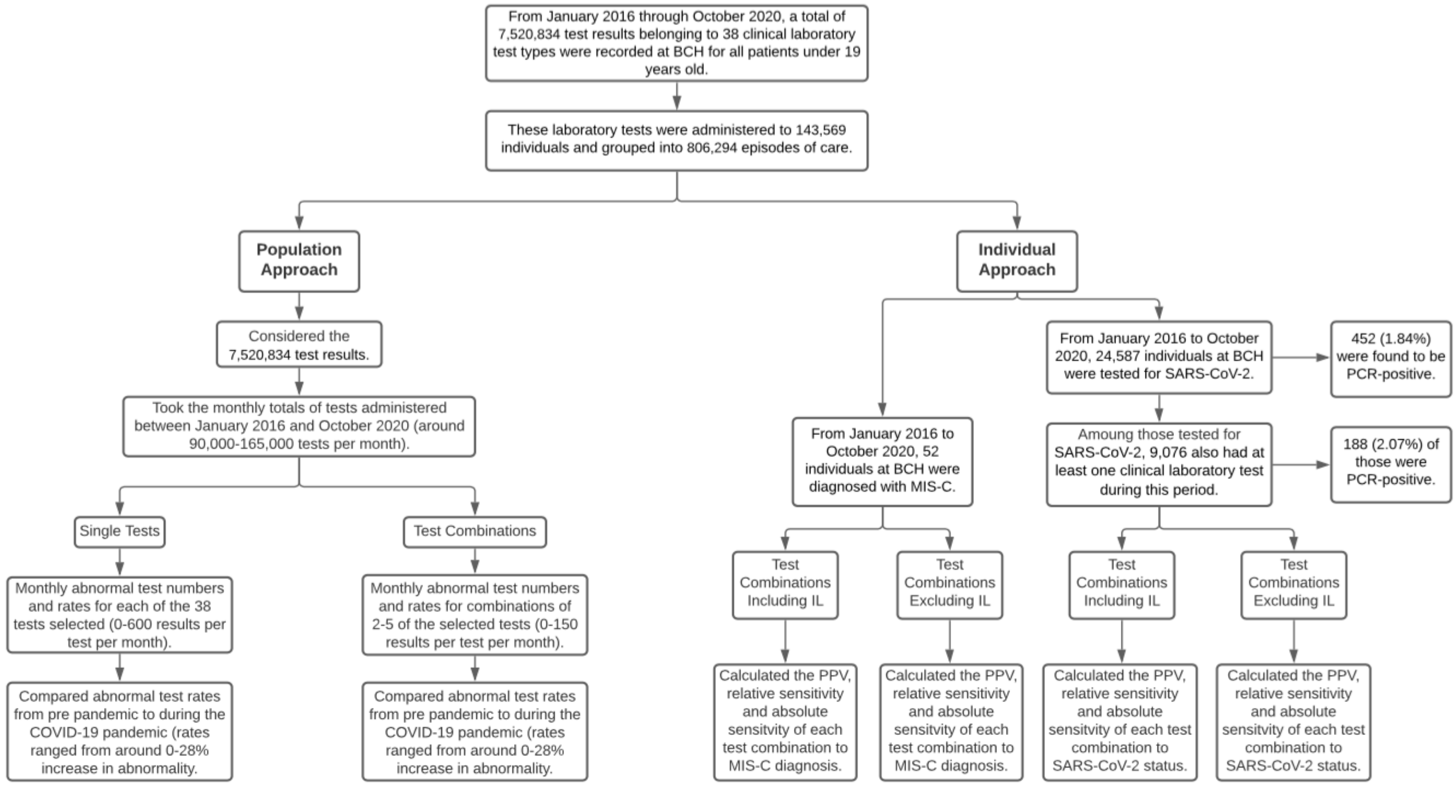

Supplementary Figure 2. **Individual Approach Overview, SARS-CoV-2:** Comparison of different laboratory test combinations, according to the absolute sensitivity (X axis) and the PPV (Y axis) for association with SARS-Cov-2 infection status. Results are shown with IL tests (top) and without (bottom).

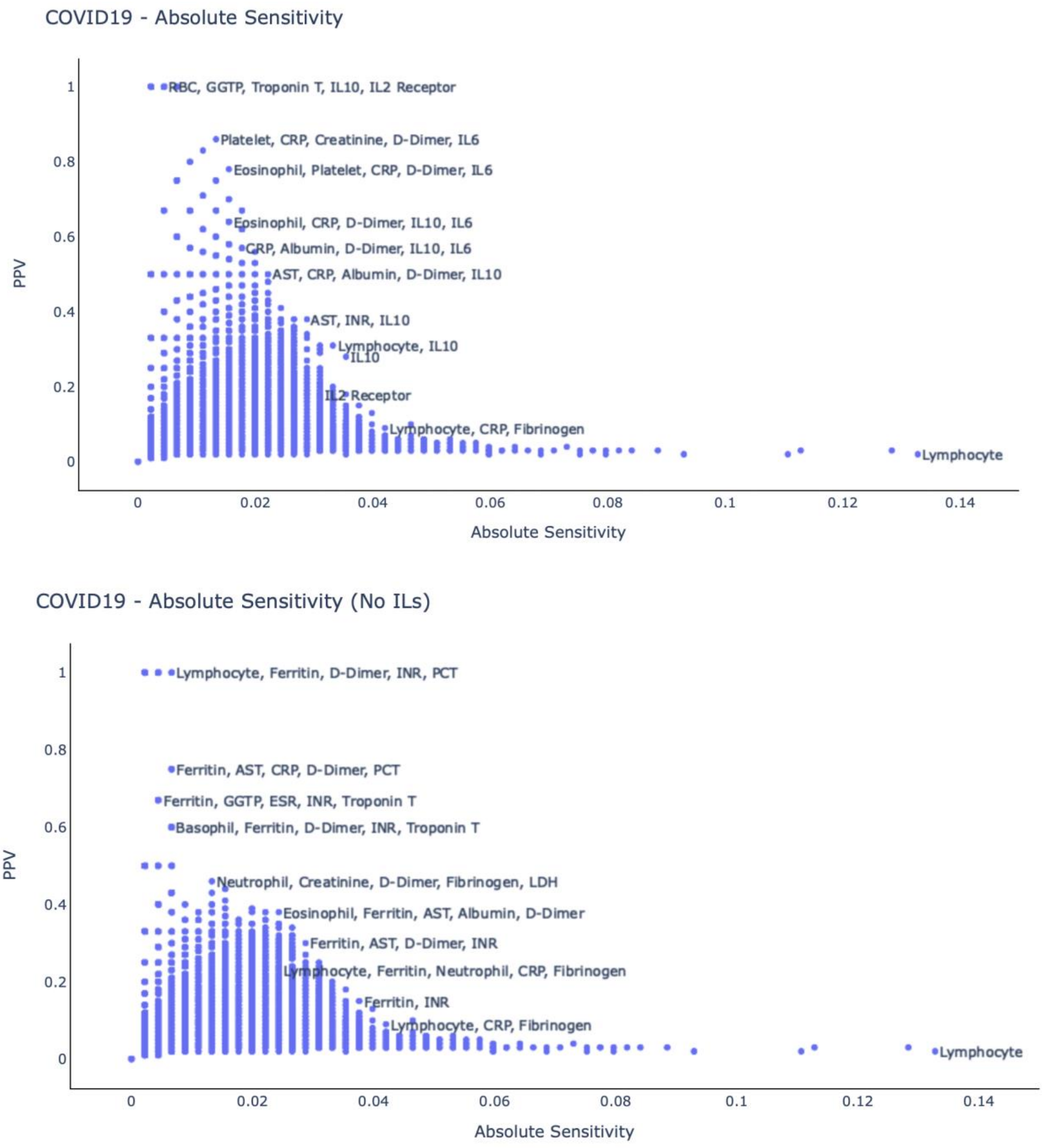

Supplementary Figure 3. Effect of Combination Size. The distribution of PPV and relative sensitivities of all observed laboratory test combinations, by increasing combination size - from 1 to 5 tests.

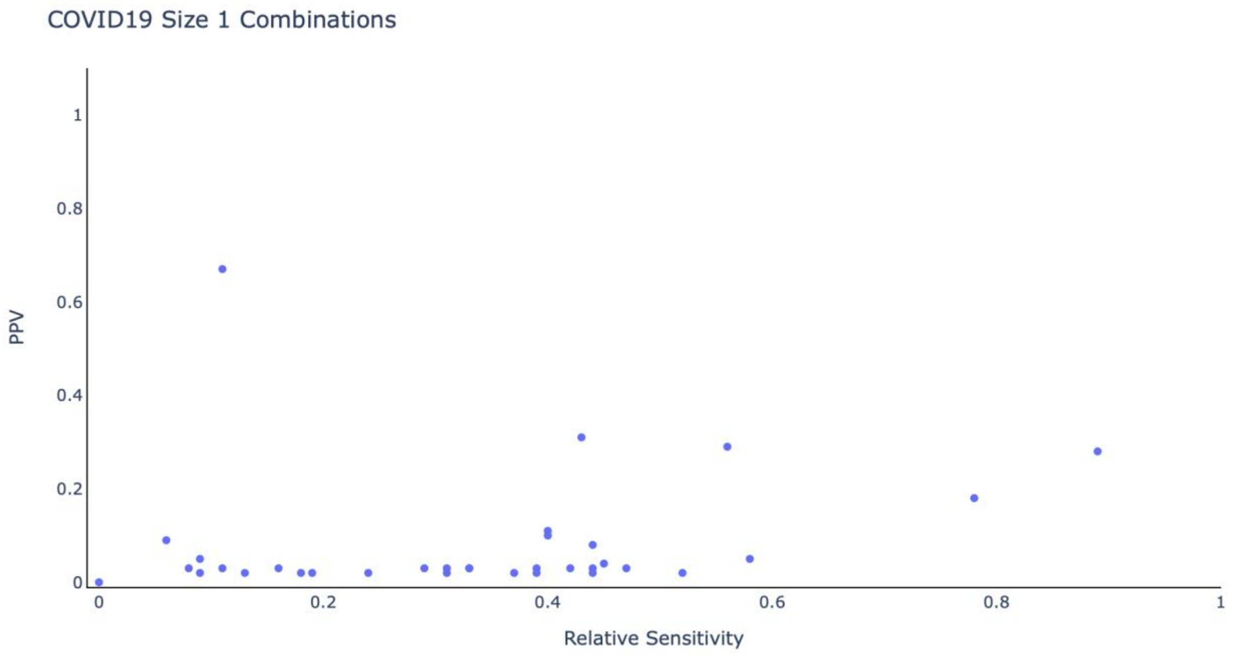

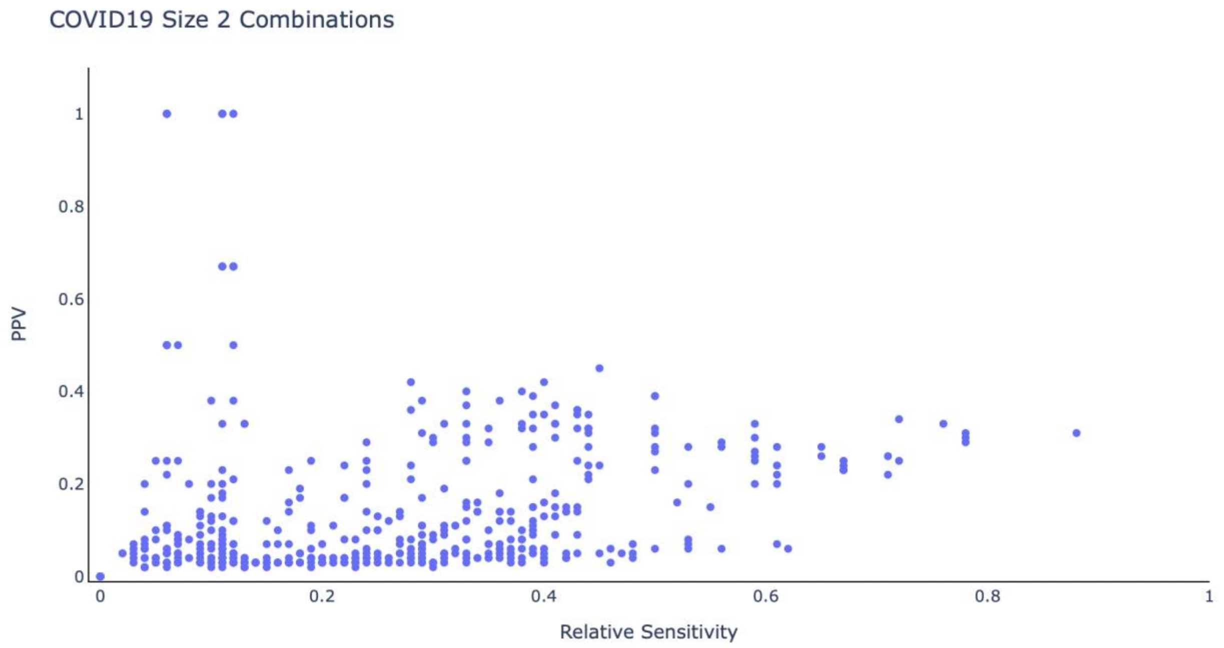

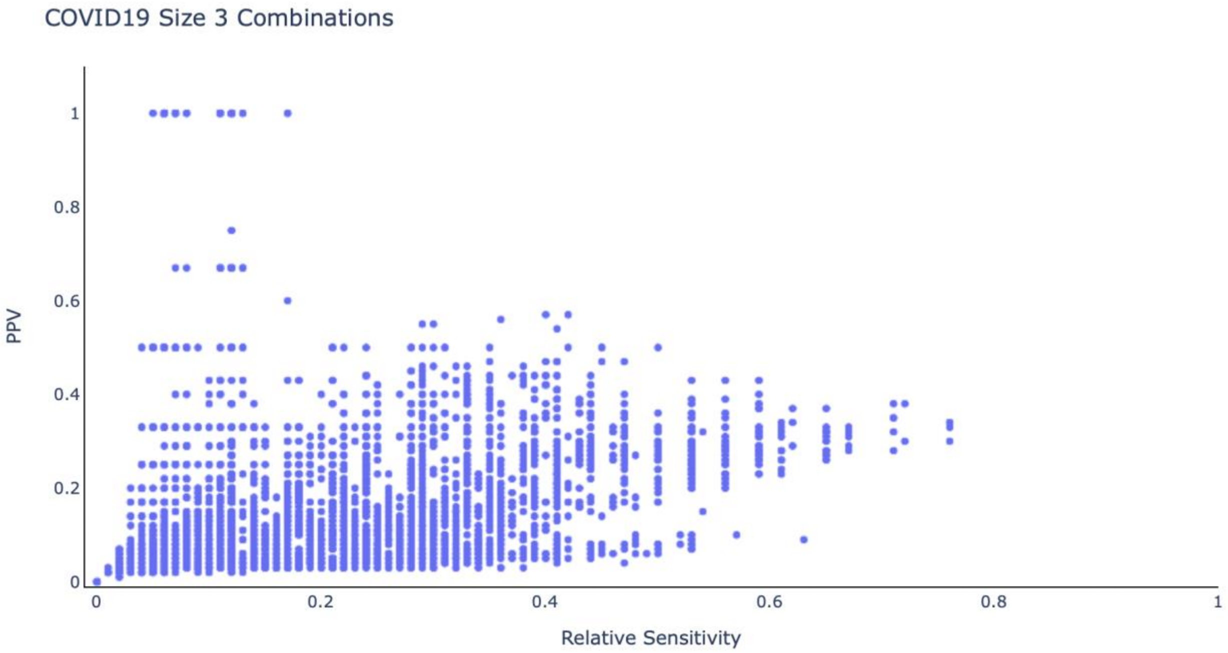
COVID19 Size 3 Combinations
PPV
Relative Sensitivity
0
0.2
0.4
0.6
0.8
1

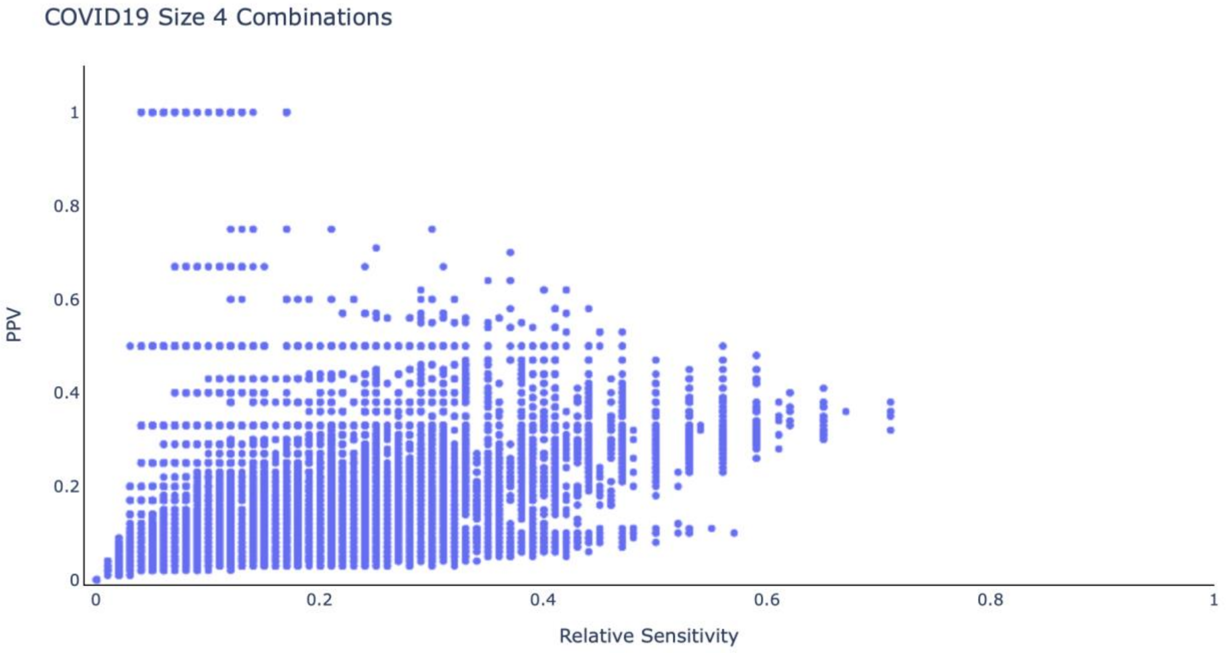
COVID19 Size 4 Combinations
PPV
Relative Sensitivity
0
0.2
0.4
0.6
0.8
1

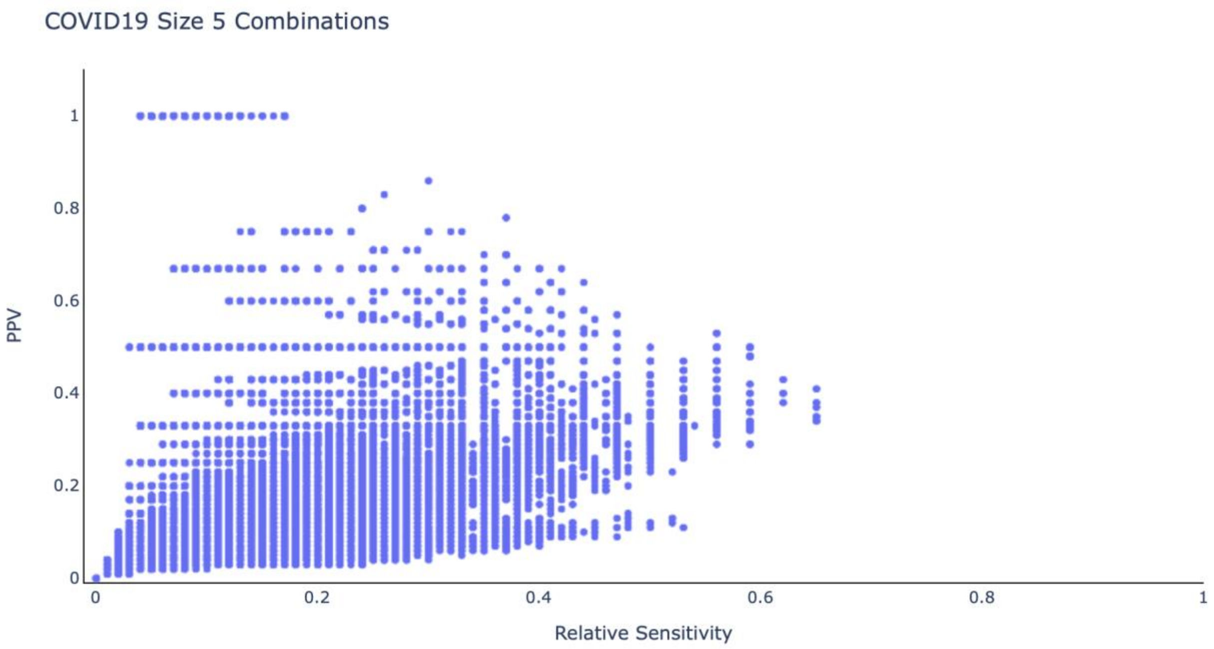

COVID19 Size 5 Combinations
PPV
1
0.8
0.6
0.4
0.2
0
0
0.2
0.4
0.6
0.8
1
Relative Sensitivity

Supplementary Figure 4. Individual Approach Overview, MIS-C. Comparison of different laboratory test combinations, according to the absolute sensitivity for MIS-C (X axis), and the PPV (Y axis). Results are shown with IL tests (top) and without (bottom).

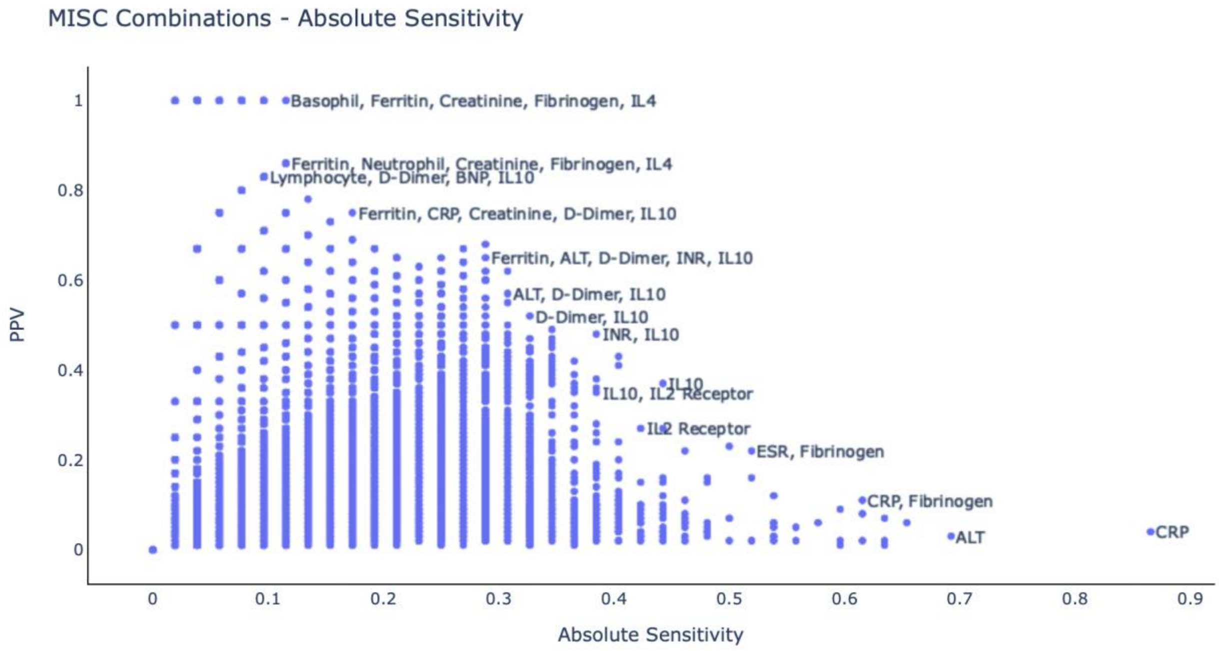

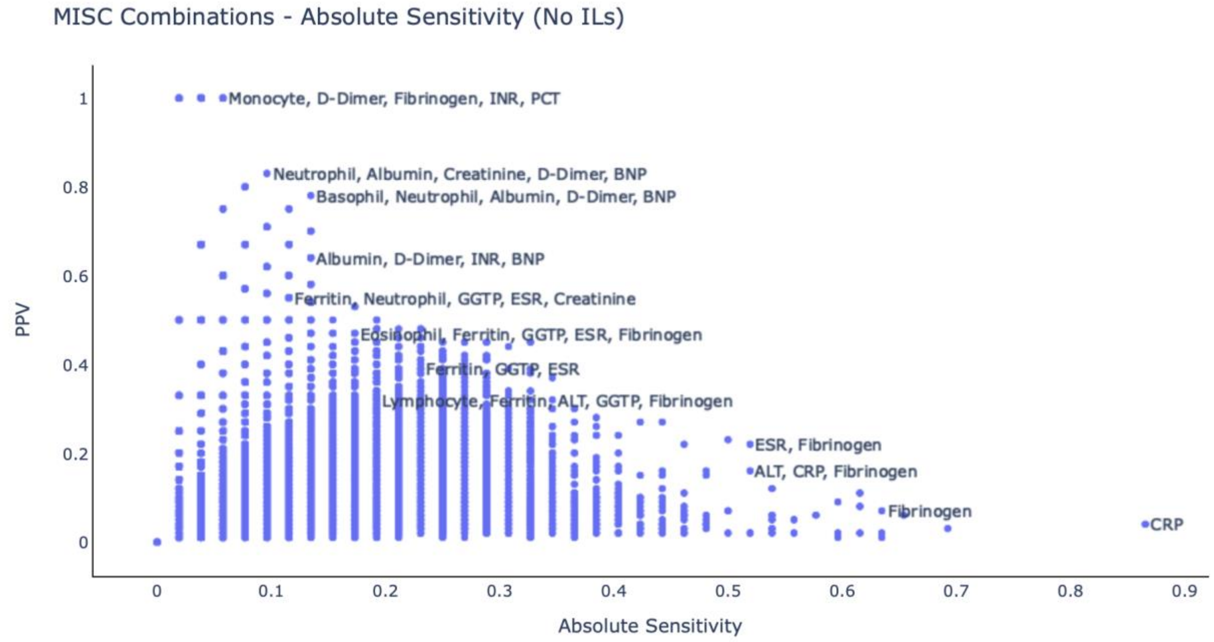

Supplementary Figure 5. **Combined Approach, SARS-CoV-2.** Comparison of different laboratory test combinations, according to the relative sensitivity (X axis) and the PPV (Y axis) for association with SARS-Cov-2 infection status. Only laboratory test combinations that peaked in April 2020 or May 2020 are shown. Results are shown for relative sensitivity for COVID-19 (top) and absolute sensitivity for COVID-19 (bottom).

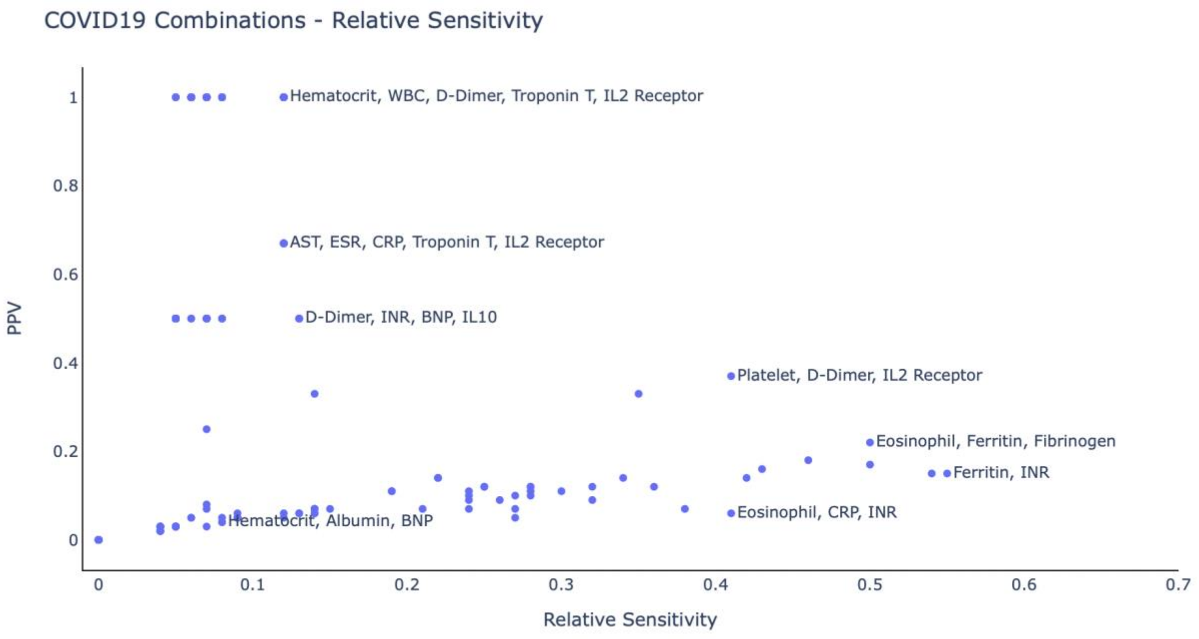

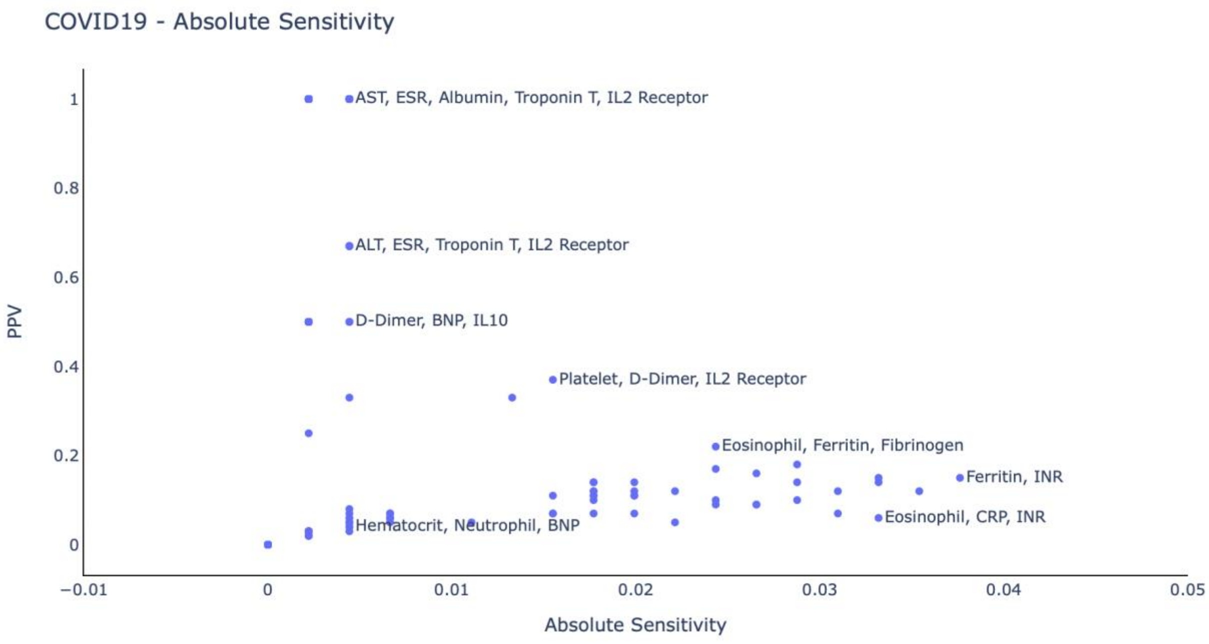

Supplementary Figure 6. Combined Approach, MIS-C. Comparison of different laboratory test combinations, according to the relative sensitivity for MIS-C (X axis), and the PPV (Y axis). Only laboratory test combinations that peaked in April 2020 or May 2020 are shown. Results are shown for relative sensitivity for MIS-C (top) and absolute sensitivity for MIS-C (bottom).

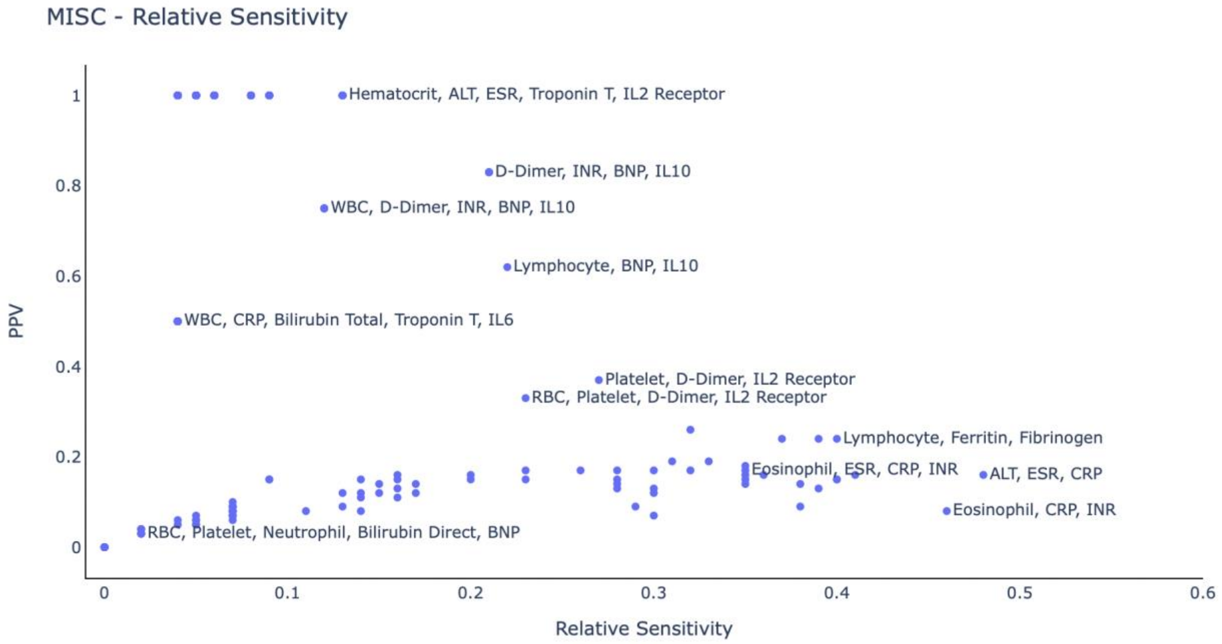

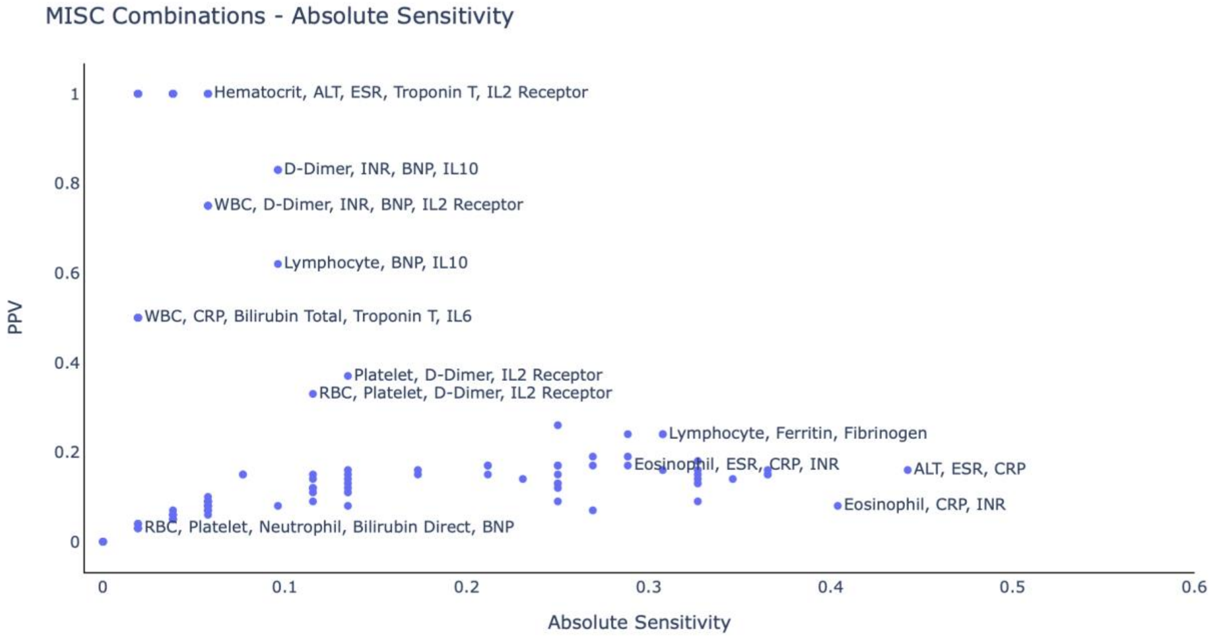

Supplementary Figure 7. Population Approach, Overview, with different sample size cutoffs. Similar to Figure 4, this plot shows a systematic comparison of all laboratory test combinations observed during the COVID pandemic, according to the number of abnormal test results (X axis), and the percentage increase in test abnormality rates relative to historical baseline (Y axis). Results are shown with Interleukin (IL) tests (top) and without (bottom).

Minimum 100

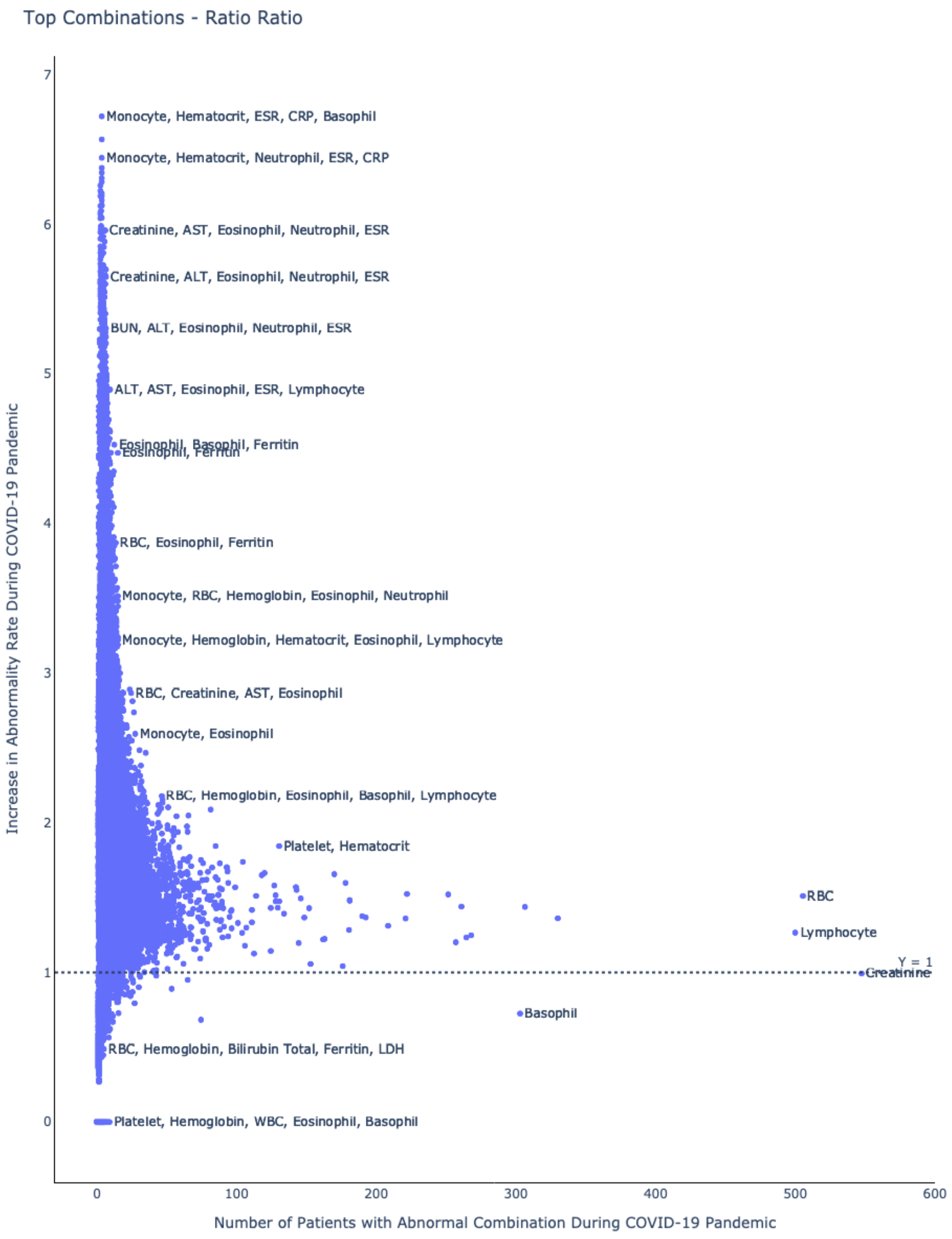

Minimum 400

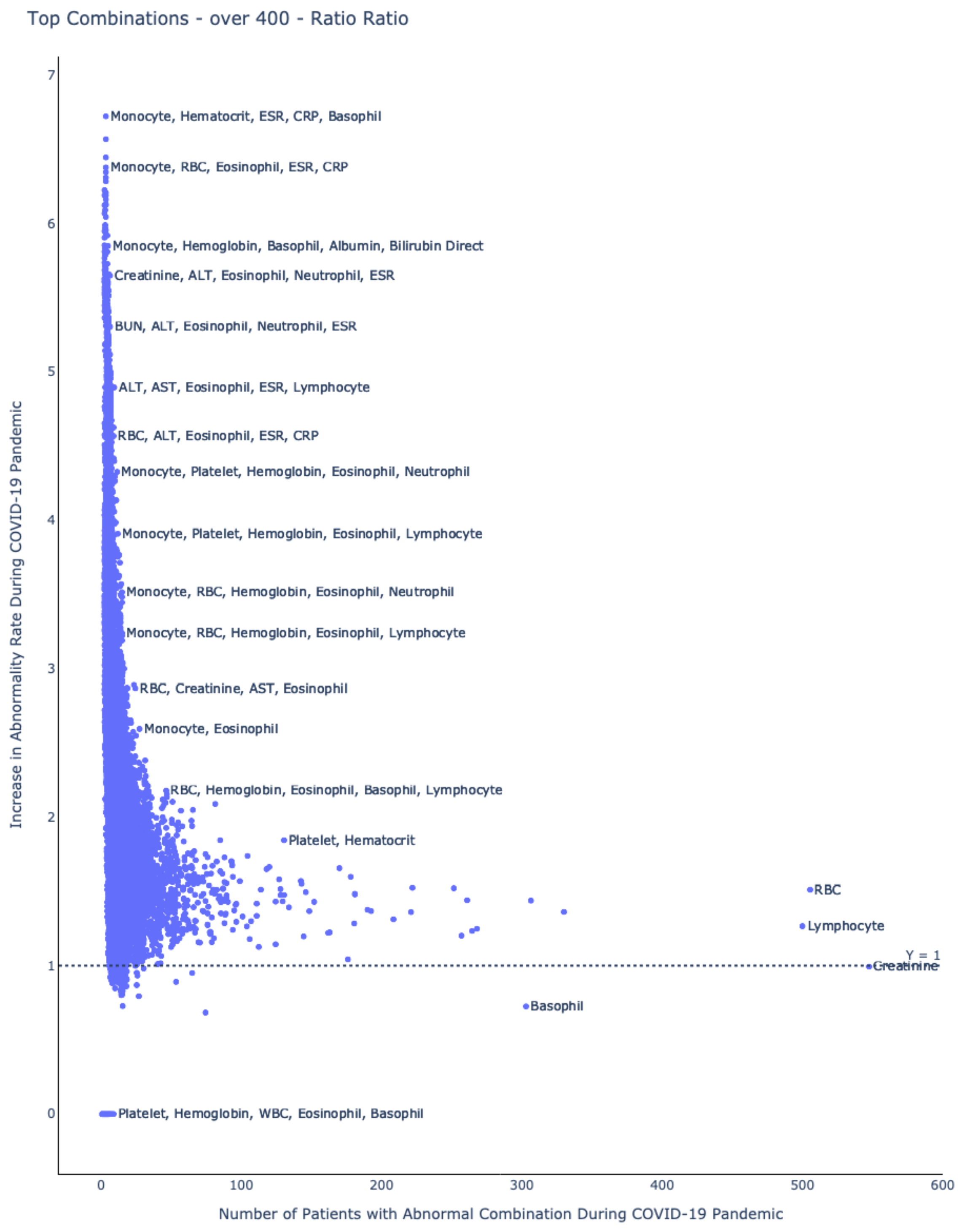

Top Combinations - over 400 - Ratio Ratio
Monocyte, Hematocrit, ESR, CRP, Basophil
Monocyte, RBC, Eosinophil, ESR, CRP
Monocyte, Hemoglobin, Basophil, Albumin, Bilirubin Direct
Creatinine, ALT, Eosinophil, Neutrophil, ESR
BUN, ALT, Eosinophil, Neutrophil, ESR
ALT, AST, Eosinophil, ESR, Lymphocyte
RBC, ALT, Eosinophil, ESR, CRP
Monocyte, Platelet, Hemoglobin, Eosinophil, Neutrophil
Monocyte, Platelet, Hemoglobin, Eosinophil, Lymphocyte
Monocyte, RBC, Hemoglobin, Eosinophil, Neutrophil
Monocyte, RBC, Hemoglobin, Eosinophil, Lymphocyte
RBC, Creatinine, AST, Eosinophil
Monocyte, Eosinophil
RBC, Hemoglobin, Eosinophil, Basophil, Lymphocyte
Platelet, Hematocrit
RBC
Lymphocyte
Y = 1
Creatinine
Basophil
Platelet, Hemoglobin, WBC, Eosinophil, Basophil
Increase in Abnormality Rate During COVID-19 Pandemic
Number of Patients with Abnormal Combination During COVID-19 Pandemic
0
1
2
3
4
5
6
7
0
100
200
300
400
500
600

Minimum 1600

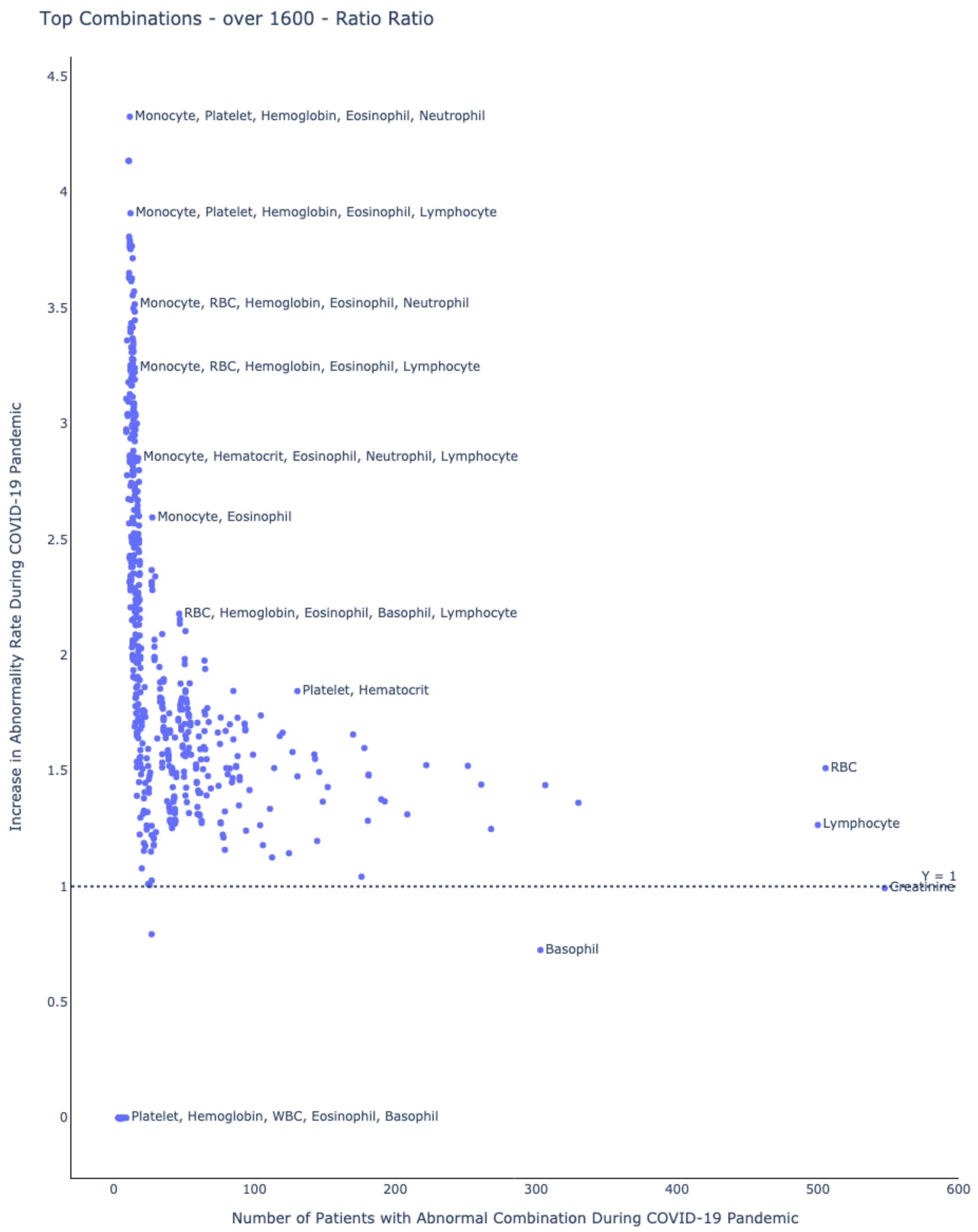

Supplementary Figure 8. **Individual Approach, SARS-CoV-2, with different sample size cutoffs.** Similar to Figure 5, this figure shows a comparison of different laboratory test combinations, according to the relative sensitivity (X axis) and the PPV (Y axis) for association with SARS-Cov-2 infection status. Results are shown with IL tests (top) and without IL tests (bottom).

Minimum 100

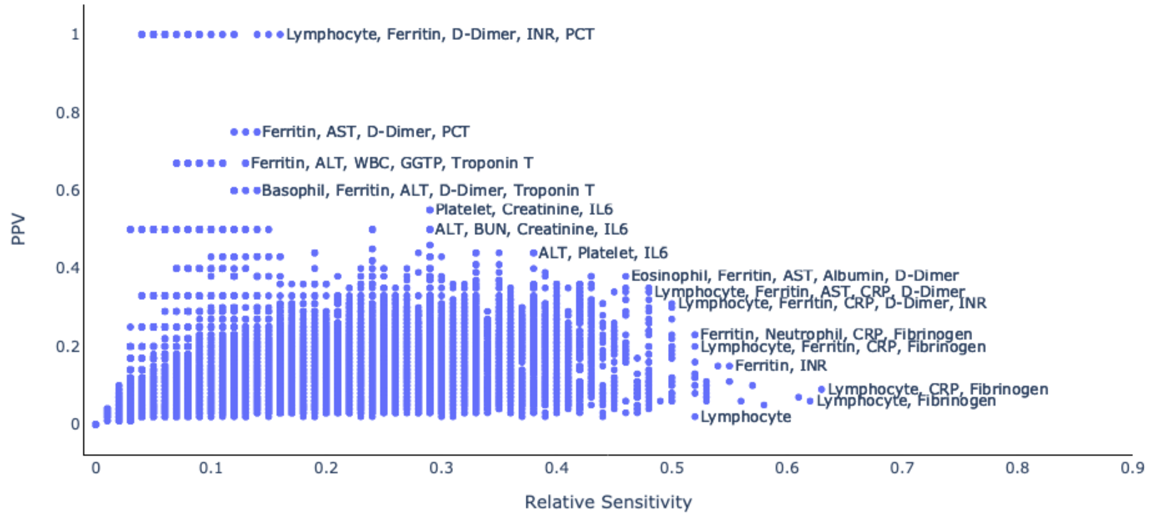

Minimum 400

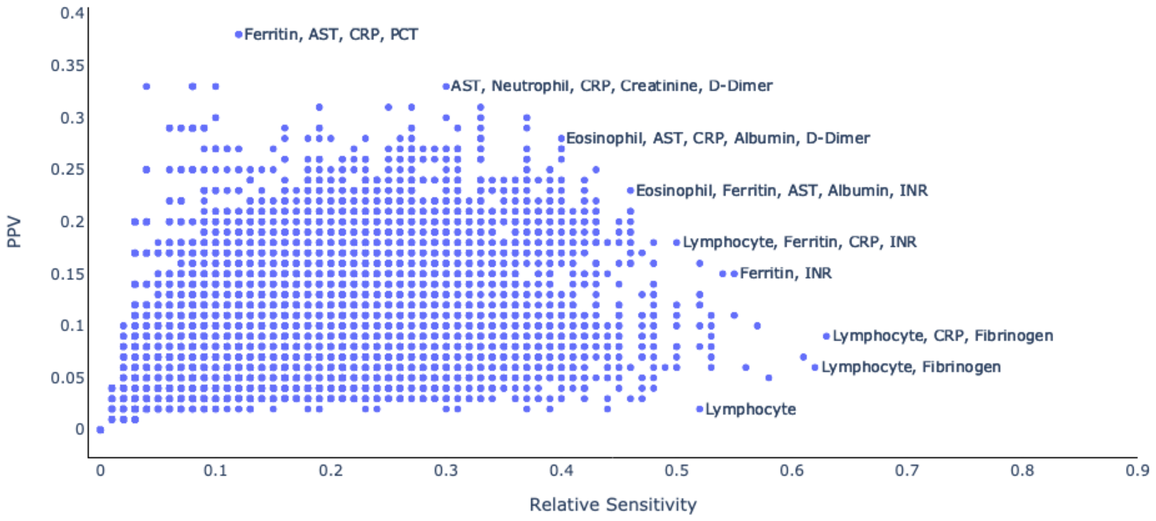

Minimum 1600

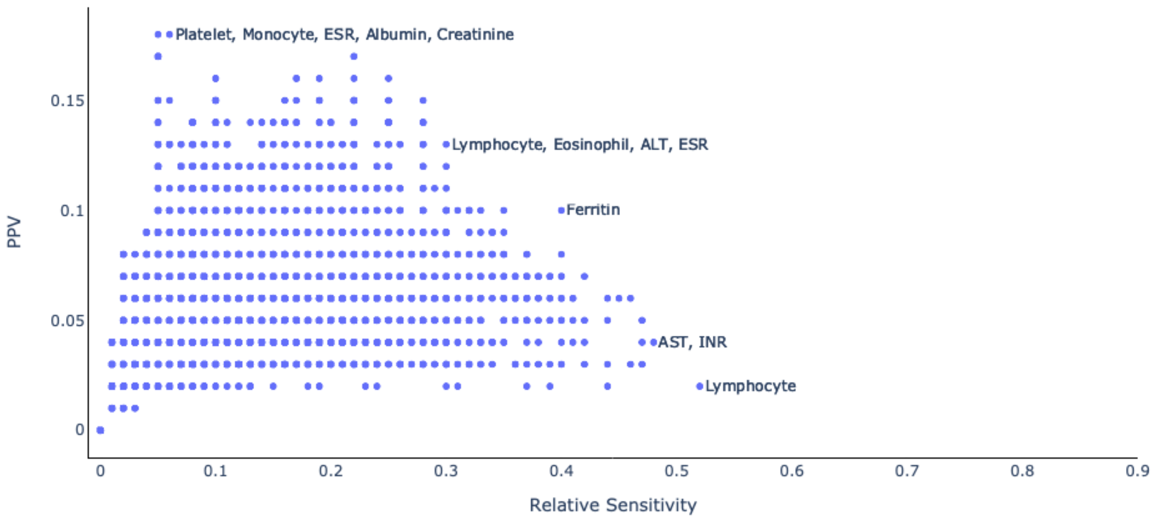

Supplementary Figure 9. **Individual Approach, MIS-C, with different sample size cutoffs.** Similar to Figure 6, this figure shows a comparison of different laboratory test combinations, according to the relative sensitivity for MIS-C (X axis), and the PPV (Y axis). Results are shown with IL tests (top) and without (bottom) IL tests.

Minimum 100

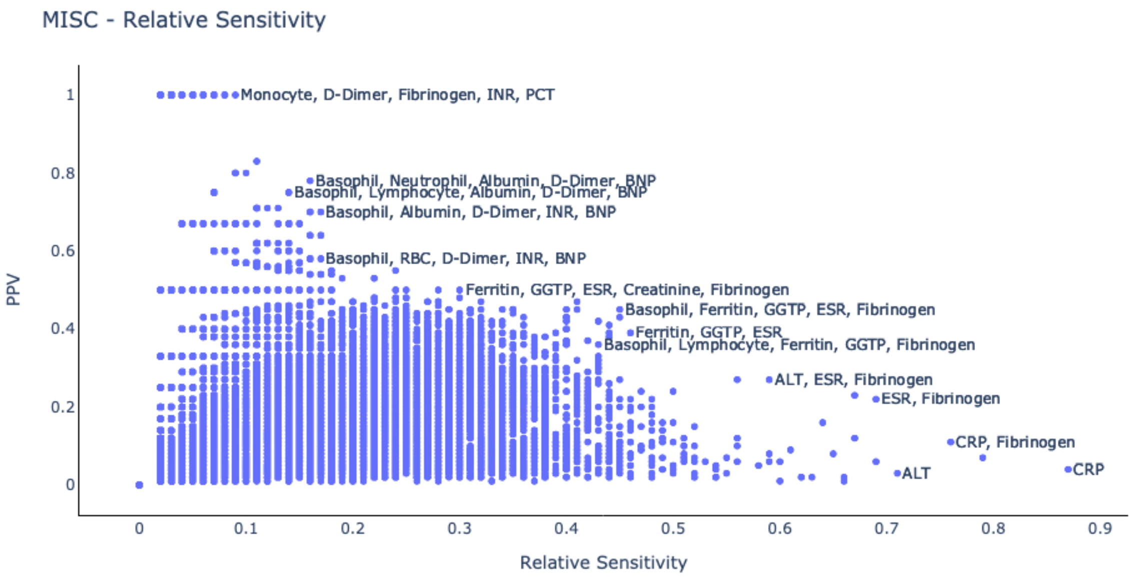

Minimum 400

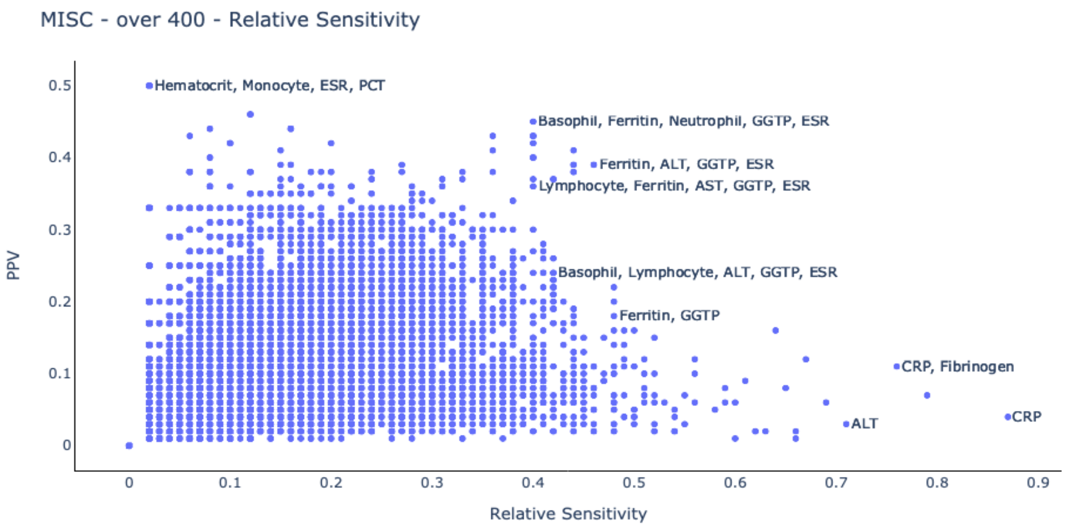

Minimum 1600

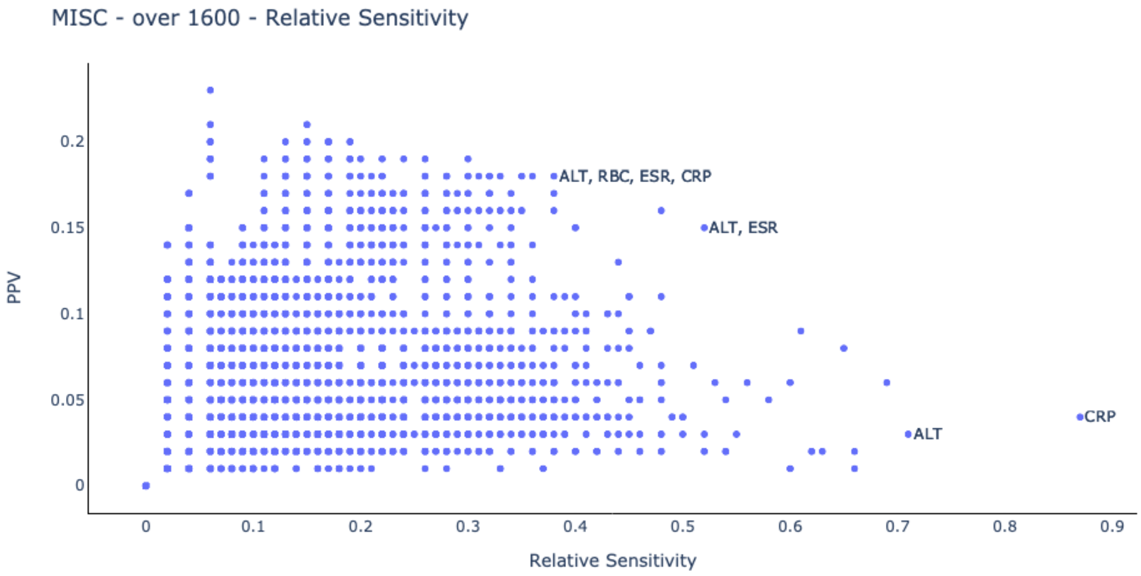

Supplement: Supplementary file 1 — Supplementary Materials [file 41746_2021_547_MOESM1_ESM.pdf]
