# Supplementary material for: An integrated framework for identifying clinical-laboratory indicators for novel pandemics: COVID-19 and MIS-C
Source: NPJ Digit Med. 2022 Jan 20;5:9. doi: 10.1038/s41746-021-00547-9 (PMC8776774; doi:10.1038/s41746-021-00547-9)
Supplement: Supplementary file 2 — Reporting Summary [file 41746_2021_547_MOESM2_ESM.pdf]

nature portfolio

Corresponding author(s): Ben Y. Reis

Last updated by author(s): 2020-10-6

Reporting Summary

Nature Portfolio wishes to improve the reproducibility of the work that we publish. This form provides structure for consistency and transparency in reporting. For further information on Nature Portfolio policies, see our Editorial Policies and the Editorial Policy Checklist.

Statistics

For all statistical analyses, confirm that the following items are present in the figure legend, table legend, main text, or Methods section.

n/a	Confirmed	
☐	☒	The exact sample size (*n*) for each experimental group/condition, given as a discrete number and unit of measurement
☐	☒	A statement on whether measurements were taken from distinct samples or whether the same sample was measured repeatedly
☐	☒	The statistical test(s) used AND whether they are one- or two-sided<br>*Only common tests should be described solely by name; describe more complex techniques in the Methods section.*
☐	☒	A description of all covariates tested
☐	☒	A description of any assumptions or corrections, such as tests of normality and adjustment for multiple comparisons
☒	☐	A full description of the statistical parameters including central tendency (e.g. means) or other basic estimates (e.g. regression coefficient) AND variation (e.g. standard deviation) or associated estimates of uncertainty (e.g. confidence intervals)
☒	☐	For null hypothesis testing, the test statistic (e.g. *F*, *t*, *r*) with confidence intervals, effect sizes, degrees of freedom and *P* value noted<br>*Give P values as exact values whenever suitable.*
☒	☐	For Bayesian analysis, information on the choice of priors and Markov chain Monte Carlo settings
☒	☐	For hierarchical and complex designs, identification of the appropriate level for tests and full reporting of outcomes
☒	☐	Estimates of effect sizes (e.g. Cohen's *d*, Pearson's *r*), indicating how they were calculated

Our web collection on statistics for biologists contains articles on many of the points above.

Software and code

Policy information about availability of computer code

Data collection	We examined data on all patients younger than 19 years old seen at Boston Children’s Hospital (BCH) between January 1, 2016 and October 31, 2020 (Table 1). We included data on 38 types of clinical laboratory tests (Table 2), SARS-CoV-2 PCR test results, and physician-confirmed MIS-C diagnoses according to the criteria defined by the Centers for Disease Control (CDC). Data collection is described in full in the Methods section.
Data analysis	We examined historical trends for the 38 clinical laboratory test types included in this study, and compared lab test results to PCR test results and MIS-C diagnoses, as described in the Methods section.

For manuscripts utilizing custom algorithms or software that are central to the research but not yet described in published literature, software must be made available to editors and reviewers. We strongly encourage code deposition in a community repository (e.g. GitHub). See the Nature Portfolio guidelines for submitting code & software for further information.

Data

Policy information about availability of data

All manuscripts must include a data availability statement. This statement should provide the following information, where applicable:

- Accession codes, unique identifiers, or web links for publicly available datasets
- A description of any restrictions on data availability
- For clinical datasets or third party data, please ensure that the statement adheres to our policy

Other than the aggregate statistics reported in the manuscript, the data analyzed for this study is restricted due to medical privacy regulations.

Field-specific reporting

Please select the one below that is the best fit for your research. If you are not sure, read the appropriate sections before making your selection.

☒ Life sciences ☐ Behavioural & social sciences ☐ Ecological, evolutionary & environmental sciences

For a reference copy of the document with all sections, see nature.com/documents/nr-reporting-summary-flat.pdf

Life sciences study design

All studies must disclose on these points even when the disclosure is negative.

Sample size	We examined data on all patients younger than 19 years old seen at Boston Children's Hospital (BCH) between January 1, 2016 and October 31, 2020 (Table 1). We included data on 38 types of clinical laboratory tests (Table 2), SARS-CoV-2 PCR test results, and physician-confirmed MIS-C diagnoses according to the criteria defined by the Centers for Disease Control (CDC).
Data exclusions	N/A
Replication	N/A
Randomization	N/A
Blinding	N/A

Reporting for specific materials, systems and methods

We require information from authors about some types of materials, experimental systems and methods used in many studies. Here, indicate whether each material, system or method listed is relevant to your study. If you are not sure if a list item applies to your research, read the appropriate section before selecting a response.

Materials & experimental systems

n/a	Involved in the study	
☒	☐	Antibodies
☒	☐	Eukaryotic cell lines
☒	☐	Palaeontology and archaeology
☒	☐	Animals and other organisms
☐	☒	Human research participants
☐	☒	Clinical data
☒	☐	Dual use research of concern

Methods

n/a	Involved in the study	
☒	☐	ChIP-seq
☒	☐	Flow cytometry
☒	☐	MRI-based neuroimaging

Human research participants

Policy information about studies involving human research participants

Population characteristics	We examined data on all patients younger than 19 years old seen at Boston Children's Hospital (BCH) between January 1, 2016 and October 31, 2020 (Table 1).
Recruitment	Retrospective analysis of de-identified electronic medical record data.
Ethics oversight	Study approved by Boston Children's Hospital IRB.

Note that full information on the approval of the study protocol must also be provided in the manuscript.

Clinical data

Policy information about clinical studies

All manuscripts should comply with the ICMJE guidelines for publication of clinical research and a completed CONSORT checklist must be included with all submissions.

Clinical trial registration	N/A
Study protocol	N/A
Data collection	Data Collection is described above and in the Methods Section.

Outcomes	Outcomes include positive PCR test for SARS-CoV-2 infection, and diagnosis for MIS-C, as described in the Methods section.
